# The multifaceted role of macrophages during acute liver injury

**DOI:** 10.3389/fimmu.2023.1237042

**Published:** 2023-09-06

**Authors:** Ghada S. Hassan, Manuel Flores Molina, Naglaa H. Shoukry

**Affiliations:** ^1^ Centre de Recherche du Centre hospitalier de l’Université de Montréal (CRCHUM), Montréal, QC, Canada; ^2^ Département de microbiologie, infectiologie et immunologie, Faculté de médecine, Université de Montréal, Montréal, QC, Canada; ^3^ Département de médecine, Faculté de médecine, Université de Montréal, Montréal, QC, Canada

**Keywords:** liver, macrophages, acute injury, wound healing response, necroinflammation, tissue repair, spatial distribution

## Abstract

The liver is situated at the interface of the gut and circulation where it acts as a filter for blood-borne and gut-derived microbes and biological molecules, promoting tolerance of non-invasive antigens while driving immune responses against pathogenic ones. Liver resident immune cells such as Kupffer cells (KCs), a subset of macrophages, maintain homeostasis under physiological conditions. However, upon liver injury, these cells and others recruited from circulation participate in the response to injury and the repair of tissue damage. Such response is thus spatially and temporally regulated and implicates interconnected cells of immune and non-immune nature. This review will describe the hepatic immune environment during acute liver injury and the subsequent wound healing process. In its early stages, the wound healing immune response involves a necroinflammatory process characterized by partial depletion of resident KCs and lymphocytes and a significant infiltration of myeloid cells including monocyte-derived macrophages (MoMFs) complemented by a wave of pro-inflammatory mediators. The subsequent repair stage includes restoring KCs, initiating angiogenesis, renewing extracellular matrix and enhancing proliferation/activation of resident parenchymal and mesenchymal cells. This review will focus on the multifaceted role of hepatic macrophages, including KCs and MoMFs, and their spatial distribution and roles during acute liver injury.

## 1 Introduction

The liver is a complex organ with multifunctional properties involved in metabolic homeostasis, protein synthesis, toxins clearance as well as immunity. Its strategic location between the gut and circulation, gives the liver a prominent surveillance function ensuring adequate tolerance to harmless antigens and immune response against invasive pathogens (1). Injuries to the liver are therefore a major health threat with severe consequences of metabolic and immune nature. In addition to describing wound healing processes in response to acute liver injuries, this article will provide an overview of hepatic macrophages, their functions and interactions with neighboring cells.

## 2 Anatomy of the liver

**Figure 1:**
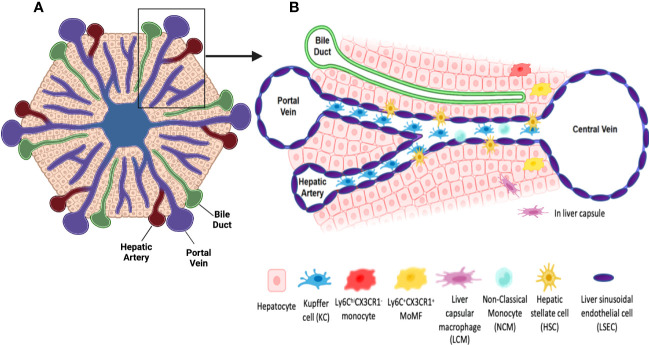
Schematic representations of a classical liver lobule and sinusoids. **(A)** The classical liver lobule has a hexagon-like structure. It is composed of a central vein surrounded by portal triads. Each portal triad is composed of a portal venule, a hepatic artery and a bile duct. **(B)** *the insert*, Central veins connect to portal veins via sinusoids which are capillary vessels where oxygen-rich blood from the hepatic artery mixes with the nutrient-rich blood from the portal vein and is delivered to the central vein. They are lined by a layer of fenestrated LSECs allowing the communication between sinusoidal blood and the perisinusoidal space of Disse and hepatocytes. KCs, the hepatic resident macrophages are located inside the sinusoids. KCs are strategically located adjacent to fenestrated LSECs to ensure clearing of portal blood from bacteria, viruses, immune complexes, effete proteins and lipids via their phagocytic activity and cytokine production. HSCs are residents of the space of Disse and exhibit a quiescent state under homeostasis. They are responsible of Vitamin A storage and metabolism. Under homeostatic conditions, few monocyte-derived macrophages (MoMFs) could be also distinguished. Liver capsular macrophages (LCMs), located in the hepatic capsule, sense and prevent the translocation of bacteria from the peritoneum into the liver. Non-classical monocytes (NCMs) patrol endothelial cells of sinusoids and are responsible for repairing vessel damage. Created using Biorender.com.

The liver is a unique anatomical, metabolic and immunological site. Its histological unit, the classical lobule, has a hexagon-like structure **(** **Figure 1** **)**. It is composed of a central vein surrounded by portal triads each of which consists of a portal venule, a hepatic artery and a bile duct. Central veins connect to portal veins via sinusoids which are capillary venules lined with a fenestrated bed of endothelial cells. Sinusoidal blood harbors immune cells capable of identifying and clearing pathogens, toxins, and cellular debris originating from the portal or systemic circulation (2). Layers of hepatocytes overlay sinusoidal capillaries encompassing the space of Disse where hepatic stellate cells (HSCs) reside (3) **(** **Figure 1** **)**. While hepatic parenchymal cells are responsible for many liver functions, non-parenchymal, mainly immune cells also undertake crucial roles in maintaining tolerance as well as providing defense mechanisms against invading agents and pathogens.

## 3 Hepatic cells

Hepatic parenchymal cells represent 70% of cells in the liver and are composed primarily of hepatocytes and also include cholangiocytes. Hepatocytes are the main cells responsible for the synthetic, secretory and detoxifying functions of the liver.

The non-parenchymal hepatic compartment is composed of different immune and non-immune cells, thus adding to the heterogeneity of liver functions. Non-parenchymal cells include the liver sinusoidal endothelial cells (LSECs) that form the lining of the hepatic sinusoidal capillaries and are in direct contact with cells in the parenchyma and those in the sinusoidal blood **(** **Figure 1** **)**. Lacking a basement membrane and harboring fenestrations with large pores (50-150 nm), LSECs regulate the flow of macromolecules and plasma in between intrasinusoidal and perisinusoidal milieus (4–6). LSECs play an important role in scavenging waste products including immune-complexes and microbial antigens (7, 8), through their exquisite machinery involving an efficient endocytosis system (9, 10) and high affinity scavenger receptors, e.g., pathogen-recognition receptors (PRRs) such as toll-like receptors (TLRs) and intracellular nucleotide binding oligomerization domain (NOD)-like receptors (11–13). They are also crucial antigen presenting cells (APCs). This antigen presenting function contributes to immune tolerance in the liver and promotes resistance to harmless antigens during homeostasis. In presence of invading pathogens and inflammatory cytokines, LSECs switch to immune activation rather than tolerance (14–18).

HSCs are another type of cells in the non-parenchymal compartment. The location of HSCs in the perisinusoidal space and their stellar shape with dendrite-like structures give HSCs the advantage of cellular contact with neighboring cells, including hepatocytes in the parenchyma, LSECs in the wall of the sinusoids and KCs in the sinusoids themselves (19) **(** **Figure 1** **)**. Under physiological conditions, quiescent HSCs store the majority of vitamin A in the body in cytosolic lipid droplets as retinyl esters, like retinyl palmitate, acetate etc. (20). Upon injury, HSCs are activated by numerous inflammatory and angiogenic mediators produced by neighboring cells (21–25) and acquire an activated phenotype. Activated HSCs (aHSCs) lose their vitamin A droplets and become proliferative, contractile and fibrogenic modulating the extracellular matrix (ECM) landscape by releasing collagen fibers, constituting as such the major cellular source of collagen deposition in the liver (26, 27). Activated HSCs also regulate the immune landscape and influence immune cells in their vicinity by releasing chemokines and cytokines (28–31) as well as growth factors (23, 32–34). While responding to chemoattractants and migrating toward the site of injury (35, 36), aHSCs are themselves a source of chemokines attracting and colocalizing with different immune cells such as monocytes and neutrophils. Indeed, studies have shown that aHSCs produce the monocyte/macrophages chemoattractants, CCL2, CCL5, CCL21 and CX3CL1 and the neutrophil chemoattractant, CXCL1 (28–31). In addition aHSCs are a source of numerous cytokines such as IL-6 and IL-10 and growth factors, e.g., hepatocyte growth factor (HGF), further impacting the hepatic immune and parenchymal cells and their response to injury (23, 32–34, 37).

The liver comprises a vast yet complementary network of immune cells responsible for inducing tolerance toward gut-derived non-pathogenic molecules while providing defensive action and immune response against antigens from invading pathogens, eliminating them and/or reducing their harmful impact. Such hepatic resident immune cells comprise innate cells and innate-like lymphocytes including dendritic cells (DCs), innate lymphoid cells (ILCs), natural killer (NK) and NKT cells, mucosal-associated invariant T cells (MAIT), and γδ T cells as well as adaptive CD4 and CD8 T cells. The liver is rich in myeloid cells, specifically macrophages that are discussed in detail below.

Finally, although not considered hepatic resident immune cells, neutrophils highly contribute to immune functions in the liver under injury conditions via an influx from circulation. Being the most abundant leukocytes in circulation in humans (70%) and mice (25%) (38, 39), neutrophils have long been considered the first immune responders of the innate immune system against extracellular bacterial or fungi infection (40, 41). While they represent a major arm of innate antimicrobial immunity, neutrophils are also capable of undertaking effector functions in inflammation and tissue damage conditions and were recently shown to actively contribute to the tissue repair response (42–44).

In summary, the liver harbors a wide range of cell types that play crucial functions at different stages of homeostasis and pathogenic conditions, this review will focus on the role of macrophages in liver disease, more specifically during acute liver injury.

### 3.1 Hepatic macrophages

The liver holds the largest pool of macrophages in the body. These cells highly contribute to hepatic immunity, and participate in liver homeostasis as well as inflammation and repair during acute and chronic injury (45). Recent development in imaging and transcriptomic techniques have revealed that hepatic macrophages are comprised of at least three different subpopulations with distinct frequencies and functions. The three main hepatic subsets of macrophages include Kupffer cells (KCs), liver capsular macrophages (LCMs), and monocyte-derived macrophages (MoMFs), with more subpopulations appearing under inflammatory conditions such as GATA6^+^ peritoneal macrophages (PRMs) and non-classical monocytes (NCMs) (45–51). Specific markers that distinguish these subpopulations in mice and humans are summarized in **Table 1**. The classical classification of macrophages as M1 and M2 cells with their pro- and anti-inflammatory signatures is no longer representative of the heterogeneity of macrophages within the liver (48, 78, 79). Recent reports have shown that hepatic macrophages could co-express markers of M1 and M2 phenotypes, thus contradicting the classical paradigm of M1 versus M2 polarization (78). Hepatic macrophages also undertake different functions in response to the plethora of signals in injured tissues rather than being purely pro-inflammatory or anti-inflammatory (80). Furthermore, the different macrophage subpopulations are present at different densities, and assume distinctive locations and functions depending on their origin and in response to signals from their environment, thus dictating their role(s) in homeostasis and response to injury (45).

**Table 1:** Frequency, Function and Markers of Hepatic Macrophages.

Hepatic macrophage population	Frequency/density during homeostasis		Function	Markers in mice	Origin	Markers in humans	References
	In mice	In humans					
KCs	20 % of CD45^+^ cells 10 KCs/100 μm^3^ of liver in C57Bl/6 mice	Not reported	**In homeostasis:** • Phagocytosis • T cell tolerance **Upon pathogen invasion or injury:** • Pathogen sensing and phagocytosis • Recruitment and activation of immune cells • Promoting tissue repair	CD45^+^ CD11b^int^ F4/80^+^ CX3CR1^-^ **Specific Marker:** CLEC4F^+^	**Yolk-sac origin:** CLEC4F^+^ MARCO^+^ Timd4^+^ Stab2^+^ CD80 (B7-1)^+^ **Bone marrow-KCs:** CLEC4F^+^ MARCO^-^ CD80 (B7-1)^++^	CD68^+^ MARCO^+^ VSIG4^+^ CD163^+^ CD5L^+^ HMOX1^+^	(19, 52–69)
MoMFs	~2 % of CD45^+^ cells	Not reported	**In homeostasis:** • Unknown **Upon injury:** • Infiltration and differentiation of inflammatory monocytes into MoMFs at the injury site. • Pro-inflammatory and pro-fibrogenic during necroinflammation • Pro-resolving during repair	CD11b^+^ F4/80^+^ Ly6C^low^ CCR2^low^ CX3CR1^hi^	**Inflammatory monocytes:** Ly6C^hi^ CCR2^+^ CX3CR1^low^	CD68^+^ MARCO^-^ CD163^low^ PLBD1^+^ LYZ^+^ CD74^+^	(48, 52, 53, 70–72)
LCMs	~ 250 cells/mm^2^ of capsule in mice	Not reported	• Bacterial sensing • Neutrophil recruitment	CD11b^+^ CD11c^+^ F4/80^+^ MHC-II^hi^ CD64^+^ CSF-1R^+^ CD14^+^ CLEC4F^-^ Timd4^-^ Ly6C^-^	Derived from adult circulatory monocytes		(49, 61, 73)
PRMs	Absent in healthy liver	Not reported	• Early infiltration of the liver injury site • Pro-repair	**Large PRMs (90%):** CD11b^hi^ F4/80^hi^ MHC-II^low^ ICAM2^+^ **Specific marker:** GATA6^+^	Embryonic progenitor or bone marrow-derived monocytes	CD14^hi^ CD16^hi^ GATA6^+^	(50, 51)
NCMs	0.23 % of CD45^+^ cells	Not reported	Surveillance and repair of the sinusoidal endothelium	Ly6C^low^ CX3CR1^hi^ F4/80^+^ MHC-II^+^ CCR2^-^		CD14^low^ CD16^+^ CD36^low^ CCR2^low^	(70, 74–77)

CCR2, C-C motif chemokine receptor 2; CLEC4F, C-type lectin domain family 4 member F; CD5L, CD5-like protein; CSF-1R, Colony stimulating factor receptor 1; CX3CR1, CX3C chemokine receptor 1; GATA, GATA binding protein; HMOX1, Heme oxygenase 1; KCs, Kupffer cells; Ly6C, Lymphocyte antigen 6 complex; LYZ, Lysozyme; MARCO, macrophage receptor with collagenous domain; MHC, Major histocompatibility complex; MoMFs, Monocyte-derived macrophages; LCMs, Liver capsular macrophages; NCMs, non-classical monocytes; PLZF, Promyelocytic leukemia zinc-finger; PRMs, Peritoneal macrophages; Stab2, Stabilin 2; Timd4, T cell immunoglobulin and mucin domain containing 4; VSIG4, V-set and immunoglobulin domain containing 4.

#### 3.1.1 Kupffer cells (KCs)

KCs are the main liver-resident macrophage subset. KCs are of yolk-sac origin and are capable of self-renewal (81–84). Despite being localized to the sinusoids, KCs interact with parenchymal cells in the perisinusoidal space via cytoplasmic processes extending through fenestrations of the sinusoidal endothelium (19, 85). Indeed, recent studies have demonstrated a high level of crosstalk between KCs and LSECs, HSCs, and hepatocytes in what is known as the KC niche, where signals from all cells within, will dictate the KC identity (19). During homeostasis, KCs do not change location. However, studies have suggested that KCs may migrate to a new location in response to several types of insults (70, 86–90). Most of these studies did not use KC-specific markers. Hence, more research into the spatial plasticity of KCs is warranted to better understand KCs and their role in hepatic immunity and response to liver injury.

Historically, murine KCs were identified as CD45^+^CD11b^int^F4/80^+^CX3CR1^-^ cells by flow cytometry and as F4/80^+^ by immunostaining. However, these markers could also be expressed on other cell types, rendering data hard to interpret. More recently, the C-type lectin domain family 4 member f (CLEC4F) was described as a specific marker of murine KCs (52, 91). CLEC4F, in combination with the markers described above, can specifically identify KCs using flow cytometry and is sufficient by itself as a marker of KCs in tissue sections (19). Murine KC subsets are also defined by their origin. Advancements in transcriptomics identified unique molecules capable of delineating murine KCs of yolk-sac origin (YS-KCs) from monocyte-derived ones. Specifically, the macrophage receptor with collagenous structure (MARCO), T cell immunoglobulin (Ig) and mucin domain containing 4 (Timd4) and stabilin 2 (Stab2) (46, 53, 92) define these KCs. This point will be discussed in more details in the following sections.

Human KCs, are identified by the expression of CD68 (93). Single cell studies in the normal human liver demonstrated the presence of two subtypes of CD68^+^ KCs, depending on MARCO expression (48). MARCO^+^ KCs preferentially reside in the periportal area, assume a tolerogenic immunosuppressive phenotype, exhibit high expression of markers associated with tolerogenic and immunosuppressive functions (e.g., V-set and immunoglobulin domain containing 4 (*VSIG4*), *CD163, CD5L^+^* (54, 61) and heme oxygenase 1 (*HMOX1*) (55) and resemble the classical murine KCs (52, 53). On the other hand, KCs negative for MARCO are enriched in the pericentral region, exhibit inflammatory functions and are rather similar to monocyte-derived macrophages (MoMFs) in mice (48, 52).

KCs participate in iron and lipid metabolism and carry multiple immune functions (56–58). They are phagocytic cells. During homeostasis, KCs perform multiple tasks, including phagocytosis of particulate material and engulfment of opsonized pathogens, aged cells, and platelets (59, 60, 94). Importantly, KCs are critical for the efferocytosis of aged neutrophils (95). This process is dependent on the IL-23/IL-17/G-CSF cytokine axis that promotes granulopoiesis in the bone marrow and clearance of senescent neutrophils by macrophages in peripheral tissues, including the liver, bone marrow and spleen (96–98). In addition, KCs contribute to the tolerogenic environment in the liver. KCs secrete IL-10 that mediates endotoxin tolerance during the physiological encounter of gut-derived lipopolysaccharide (LPS) (99). During the steady state, KCs also mediate T cell tolerance against commensal microbes and dietary antigens upon exposure through the oral route. Furthermore, KCs are important APCs as they process and present antigens in the context of both major histocompatability complex (MHC)-I and MHC-II, and express costimulatory molecules and are thus capable of priming naïve CD4 and CD8 T cells. This antigen presenting capacity also contributes to their tolerogenic function as KCs mediate the differentiation of CD4 T cells into regulatory T cells (T regs) and promote CD8-induced deletional tolerance during homeostasis (62, 63, 100, 101). Finally, KCs are protective against T cell-mediated allograft rejection following liver transplantation (101–103).

In summary, KCs enhance tolerance under static conditions. Given their enrichment in the periportal regions, KCs are capable of rapidly detecting pathogenic exposures, where they exhibit pro-inflammatory functions and orchestrate the hepatic immune response. Their distinct roles in liver injury will be further discussed in the following sections.

#### 3.1.2 Liver capsular macrophages (LCMs)

Another subset of liver-resident macrophages, the LCMs, form a network of cells in the hepatic capsule. LCMs are dendritic-like cells that express various macrophage markers on their surface including F4/80, CD64, CSF-1R, and CD14, but lack the specific markers of KCs (CLEC4F and Timd4) and of monocytes (Ly6C) (49, 61, 73). In addition, while KCs originate from embryonic yolk-sac stem cells, LCMs are derived from circulating MHC-II^+^ monocytes (49), defined as the intermediate subset of monocytes (104). Located in the hepatic capsule, LCMs sense and prevent the translocation of bacteria from the peritoneum into the liver. They were shown to promote the influx of neutrophils in infections with *Listeria monocytogenes* as well as *Mycobacterium tuberculosis* (49).

#### 3.1.3 Monocyte-derived macrophages (MoMFs)

MoMFs are classified as emergency responders, however they could be found in normal livers. In mouse liver under homeostasis, MoMFs are scarce and constitute only a small percentage of hepatic macrophages (45, 53, 70, 71). In mice, hepatic MoMFs originate from bone marrow-derived circulating Ly6C^hi^CCR2^+^CX3CR1^low^ inflammatory monocytes (53, 71, 78). Indeed, under pathological liver conditions due to pathogenic infections or liver injuries, KCs, HSCs and LSECs release various cytokines and chemokines, such as CCL1 and CCL2, inducing the influx and activation of bone marrow-originated circulatory monocytes (35, 105–107). At the injury site, recruited inflammatory monocytes then undertake a phenotypic switch into macrophages and become Ly6C^low^CCR2^+^CX3CR1^hi^ (53, 71, 78). The various functions of MoMFs during liver injury will be discussed below.

## 4 Immune responses during acute liver injury

### 4.1 Acute liver injury

Acute liver injury (ALI) is the main risk factor for acute liver failure in humans. It is caused by multiple aetiologies including drug-induced hepatotoxicity, autoimmunity and viral hepatitis (108). ALI is mainly manifested as increased levels of alanine aminotransferase (ALT) and aspartate aminotransferase (AST). ALT catalyzes the formation of pyruvate and glutamate as part of the alanine cycle. AST, on the other hand catalyzes the reversible conversion of aspartate to glutamate and is thus also implicated in amino acid metabolism. Both enzymes are highly present in hepatocytes and leak onto the circulation upon hepatocellular injury (109). Liver transplantation constitutes the major therapeutic option of acute liver failure. For drug-induced acute liver injury, mainly caused by acetaminophen overdose and the release of highly oxidative metabolites that provoke hepatotoxicity (110), N-acetyl cysteine (NAC) has been widely used as a therapeutic agent (111). Its strong anti-oxidant properties allowed NAC not only to be the drug of choice for drug-induced liver injury but also to be used in the treatment of other diseases (112–114). However, the high prevalence of ALI, the persistence of risk factors, and the scarcity of transplantation organ supply and treatment options urge for alternative therapeutic strategies and call for deeper investigations into mechanisms of disease pathogenesis. In this context, several rodent models of ALI were developed. **Table 2** summarizes these models, their injury mechanism, their pros and cons or limitations. Liver injury animal models include toxin-induced [e.g., acetaminophen, D-galactosamine, or carbon tetrachloride (CCl_4_)], immunological (e.g., concanavalin A-, anti-Fas- antibody, or hepatitis virus-induced), and surgical (hepatectomy or portocaval anastomosis and hepatic artery ligation) models. Depending on the objective of the study, the features of the injury (acute versus chronic) and the clinical correlate in question, one model could be favored over others (see pros and cons columns in **Table 2**). Upon injury, a dynamic sequence of cellular events occurs to contain the damage and repair the tissue dysfunction in a process termed the wound healing response.

**Table 2:** Rodent models of acute liver injury.

Category	Model	Mode of Action	Pros	Cons	References
**Toxin-induced**	Acetaminophen (APAP)	Cytochrome P450-dependent hepatotoxic metabolite NAPQI	• Recapitulates AHF following acetaminophen overdose • Dose dependent effect	• Inducing cytochrome P450 or depleting glutathione may be needed for proper disease presentation • Less effective in rats as compared to mice	(115–119)
	D-galactosamine	Uridine depletion Given with or without lipopolysaccharide	• Histologically and biochemically compatible with AHF • Efficient for studying renal damage and hepatic metabolic dysfunction in AHF	• No direct corresponding clinical condition • Distinctive histological presentation compared to other toxins.	(120–124)
	Carbon tetrachloride	Cytochrome P450 dependent highly reactive radicals	• Used to study generalized hepatotoxicity • Also used as a chronic liver injury model	• No clinical analogue to this chemical • Poor model for hepatic encephalopathy • Variable susceptibility in between species	(125–128)
**Immunological**	Concanavalin A	Immunogenic lectin	• T cell-mediated liver injury • Highly representative of Autoimmune hepatitis	• Age, sex and strain differences in susceptibility	(124, 129–131)
	Anti-Fas antibody	Fas-mediated cell killing	• Used to study cell death by apoptosis in AHF and ALF	• Off-target effects in non-hepatic tissues (*e.g*., spleen, thymus) • Mouse strain differences in susceptibility	(124, 132)
	Viral	Endemic, species-specific or genetically modified viruses	• Important clinical cause of ALF • Low transmission rate of species-specific viruses to research personnel	• Difficult to induce using human viruses	(133, 134)
**Surgical**	Partial hepatectomy (Resection of 70–97% of liver)	Left, middle and partial right lobes resection	• Equivalent to large liver resections (after cancer diagnosis) • Recommended for studying liver regeneration	• Does not reproduce complications usually seen in clinical ALF including hepatic encephalopathy • Specific to liver failure following hepatectomy	(124, 133, 135–137)
	Portocaval anastomosis and hepatic artery ligation	Two procedures done simultaneously or separately	• Reproducible • Useful to study liver failure following ischemia	• Could be irreversible (depending on the time of hepatic artery occlusion)	(133, 138–140)

NAPQI, N-acetyl-p-benzoquinone imine; AHF, Acute hepatic failure; ALF, Acute liver failure.

### 4.2 Advanced techniques for the assessment of the response to liver injury

Investigations of the immune response to injury initially involved analyzing whole cell populations and comparing their profiles and biological activity under a given pathological condition with respect to physiological states. In the last two decades, new high throughput imaging and transcriptomic technologies were developed and used to study intrahepatic immunity. Advances in flowcytometry like the use of cytometry by time of flight (CyTOF) allowed the use of a high number of markers for better phenotypic characterization of intrahepatic cells (92). Technologies investigating gene expression profiles evolved from bulk transcriptomics to higher resolution techniques involving single cell evaluations (single-cell RNA sequencing (scRNA-seq), single-nucleus RNAseq (snRNA-seq), and assay for transposase-accessible chromatin with sequencing (ATAC-seq) (61, 141). This revolutionized studies at the single cell level and revealed the importance of these techniques in delineating specific cell populations at play and in overcoming the heterogeneity within and between diseased tissues. The use of these technologies in understanding liver physiology and disease are reviewed elsewhere (142).

Additional advancement in these technologies involved combining single-cell profiles with histological information and allowed a better understanding of the spatial-functional characteristic of cells within normal or diseased tissues. Such technologies include histo-cytometry, imaging mass cytometry (a combination of mass spectrometry with laser ablation, using metal-coupled antibodies that allows multiplex imaging of numerous markers within the same tissue section), iterative bleaching extends multiplexity (IBEX), spinning-disk confocal intravital microscopy (SD-IVM), and CO-Detection by indEXing (CODEX, a technology utilizing antibodies conjugated to unique oligonucleotide barcodes) (71, 143–149). In the same context, advances also included spatial transcriptomics where single cell transcriptomics are combined with histological information allowing for better mapping of cells with resolved gene expression (e.g., RNAscope and Visium Spatial Gene Expression) (61, 150).

However, these technologies, while providing highly specific and pertinent data in the field of immune responses including the interesting relationship between spatial distribution and function of immune cells in health and disease, they require the use of expensive equipment and/or software and could not thus be available to all researchers. In addition, the multitude of markers needed for the identification of hepatic cell populations and their subsets represent a limitation to some of these approaches. To overcome some of these challenges, our group developed an accessible and feasible technique capable of providing overall tissue distribution of different cell populations. We utilized a strategy that combined serial/sequential labeling and multiplex IF with modern image analysis techniques including tissue alignment, tissue segmentation, automated quantification of cells of interest and tissue heat mapping. This allowed the detection, quantification, and spatial plotting of multiple immune cell populations simultaneously in the hepatic tissue and at the injury site (151).

In summary, recent advances in the investigation of immune cells in various types of liver pathologies have allowed a better understanding of the heterogeneity of these cells reflected by their distinctive spatial distribution and biological function. In the subsequent sections, we will focus on the spatial interactions of the immune cells during acute hepatic injury.

### 4.3 Wound healing response to acute liver injury

The wound healing response is an orchestrated process that involves resident and infiltrating immune cells interacting with tissue cells in response to injury and aiming at restoring homeostasis. Upon acute liver injury, the first phase of the wound healing response is necroinflammation and involves hemostasis and immune cell infiltration in response to tissue damage. The subsequent stage, termed repair phase is characterized by angiogenesis, renewal of ECM and proliferation of resident parenchymal cells (152, 153).

#### 4.3.1 The Necroinflammation phase of the wound healing response

Necroinflammation is a process that involves necrotic death of tissue cells inducing an inflammatory wave. It could be detected as early as 6-12 hrs post-injury depending on the injury type. For instance, we recently showed in a model of CCl_4_-induced acute liver injury, that necroinflammation encompasses a time frame from 12-48 hrs (70). In the acetaminophen (APAP)- injury model, necroinflammation is initiated at 6 hrs after the insult up to 48 hrs when the hepatic necrosis markers, ALT and AST begin to normalize (154, 155). Similar time courses of necroinflammation and the subsequent repair phase was demonstrated in numerous studies with other acute liver injury models (156).

Necrosis in this phase is usually induced by a regulated programmed cell death process. Damage-associated molecular patterns (DAMPs) released from dead cells activate the inflammation process and promote the initiation of subsequent repair functions. Indeed, abnormal inflammatory and auto-immune responses were shown to result from defective necrosis-inducing pathways (153, 157). However, a non-regulated form of cell death has also been reported and termed traumatic necrosis. In toxin-induced liver injury, endogenous enzymes act on these toxins and catalyze the generation of noxious free radicals that promote necrotic cell death. These enzymes are highly expressed and have stronger activity in hepatocytes of the pericentral area, reflecting the importance of zonation at the level of liver damage (158). In the same line of evidence, zonated necrosis was demonstrated in central veins in CCl_4_-, APAP- or thioacetamide (TAA)-induced acute liver injury (70, 159–161).

Inflammation during this stage is initiated by an immune imbalance characterized by the activation of resident cells that secrete cytokines and chemokines and promote the recruitment of circulating immune cells. At the same time, activated cells may die or differentiate into other subsets.

##### 4.3.1.1 Lymphocytes in necroinflammation

In the necroinflammation phase, a reduction of the resident lymphoid population is observed. Our group demonstrated decreased numbers of NKT cells, CD4 and CD8 T cells, and B cells in the early stages post-injury and their return to baseline levels in the repair phase (70). However, in the severe combined immunodeficient mice (SCID) mouse model that lacks mature T and B cells, CCl_4_ treatment resulted in lower necrosis in the injured liver with low hepatocellular death and ALT levels, suggesting a role for T or B cells in tissue damage. Similar data marking the tissue-damaging effect of T cells but not B cells were reported in other injuries, such as those induced upon ischemia reperfusion, using T cell-deficient mice (CD3ϵKO) and B cell-deficient ones (muMt-) (162). These data, and given the scarce presence of T cells reported in some CCl_4_-injury studies during necroinflammation (70), suggest that the involvement of T cells in the wound healing response to CCl_4_ injury might be restricted to the sensing of damage and early amplification of the inflammatory response.

##### 4.3.1.2 KCs in necroinflammation

We and others have demonstrated that the number of KCs is decreased significantly to approximately 25% of the steady state KC population during the necroinflammation stage in the CCl_4_-acute injury model (70) and other liver injury models (46, 53, 163, 164). The reduction in the number of KCs has also been reported in the early inflammatory phase of most models of liver injuries, as reviewed elsewhere (54). The injury site is repopulated later during the repair phase through proliferation of remaining KCs (53, 70), where they colocalize with aHSCs, suggesting their resolving potential.

KCs also exhibit an inflammatory signature in the response to liver injury. They detect pathogenic and danger signals from injured cells, i.e. hepatocytes or LSECs via their large repertoire of PRRs, including TLRs, NOD-like receptors, and retinoic acid-inducible gene-I (RIG-I)-like receptors and release numerous inflammatory chemokines and cytokines initiating the infiltration and activation of other immune cells at the injury site (60, 165, 166). Activated KCs promote recruitment of myeloid cells namely, monocytes and neutrophils to the injury site via the release of CCL2, CXCL1, and CXCL2 (35, 64, 164). In addition, upon liver injury, TNF-α, mainly derived from activated KCs (167) as well as DCs (168, 169) contributes to the secretion of other pro-inflammatory cytokines and chemokines including IL-1α, IL-6, CCL2, CCL3, and CCL4, further allowing recruitment of monocyte/macrophages to the injury site (70, 168, 169). It is important to mention here the inverse relationship reported between TNF-α release and IL-10 levels, whereby lack of IL-10 was shown to associate with enhanced neutrophil influx and TNF-α levels in CCl_4_-induced models (170, 171) and treating LPS-stimulated KCs with IL-10 *in vitro* decreased their release of TNF-α (172). Another evidence that underscores the pro-inflammatory role of KCs in liver injury, is their release of IL-1β, a key player in liver damage. IL-1β derived from activated or dead cells, stimulates pro-inflammatory responses in neighboring cells enhancing their expression of adhesion molecules such as intercellular adhesion molecule-1 (ICAM-1) on mesenchymal cells and vascular cell adhesion molecule−1 (VCAM-1) on endothelial cells, as well as iNOS and chemokines and promoting influx of inflammatory and immune cells from circulation into the injury site (173, 174). Similarly, IL-1β receptor-deficient mice demonstrated reduced necrosis upon their challenge with TAA (175).

##### 4.3.1.3 Inflammatory monocytes in necroinflammation

The influx of circulating cells to the injury site and their activation is a characteristic feature of the necroinflammatory phase. This is especially evident by inflammatory monocytes (53, 71) that peak during necroinflammation and locate to the perimeter of the injury (70, 71). Upon focal thermal liver injury, inflammatory monocytes (Ly6C^hi^CX3CR1^low^) are recruited to the liver very early during the response and later on differentiate into MoMFs during the transition from necroinflammation to repair (71). Such phenotypic switch is dependent on IL-4, IL-10, CX3CL1, neutrophil-derived ROS, and phagocytosis of dead neutrophils (28, 44, 71, 78, 176). Using the CCl_4_-injury model, we observed a comparable course of influx/differentiation of these cells during similar phases of the wound healing response. Indeed, our data showed MoMFs to be at their highest level following the peak infiltrate of monocytic cells, i.e. at the early repair phase (70). Studies using the APAP-induced liver injury model also revealed large numbers of MoMFs in necrotic areas around central veins at the transition from necroinflammation to repair (177). Finally, a pro-restorative gene signature was reported for MoMFs during the repair phase, suggesting an active role for these cells in alleviating necrosis and reducing tissue damage (53). MoMFs and their functions will be discussed in detail in the repair phase section below.

##### 4.3.1.4 Neutrophils in necroinflammation

Neutrophils are the most abundant leukocyte type in circulation. They are important players in inflammation and tissue necrosis upon liver injury. They are capable of capturing pathogens via complement receptors, Fc receptors, integrins or neutrophil-extracellular traps (NETs) and of eliminating them by phagocytosis, by the release of granules containing microbicidal molecules, reactive oxygen species, lysing enzymes or by NETosis (42). While few neutrophils are present in the liver under homeostatic conditions, their frequency and distribution are altered during injury as reported in many models. They are recruited at the injury site in response to chemoattractants such as CXC chemokines including CXCL1, CXCL2 released from activated monocytes/macrophages and KCs (43). They were also shown to induce tissue damage and impairment in numerous acute and chronic injury conditions (42–44). It is important to note that while acute liver injury induced by different doses of APAP promoted the influx of neutrophils into the liver, the contribution of these cells to the injury was revealed at higher APAP doses (178).

##### 4.3.1.5 Non-classical monocytes (NCMs) in necroinflammation

NCMs are identified in murine models as Ly6C^low^CX3CR1^hi^F4/80^+^MHC-II^+^. Human NCMs are CD14^low^CD16^+^CD36^low^CCR2^low^, representing 2-11% of circulating monocytes (74–77). They have been characterized as patrolling cells that protect against vascular injuries and are elevated during liver inflammation (70). Studies demonstrated that NCMs patrol healthy tissue and engage in rapid tissue invasion in a CX3CR1-dependent manner upon exposure to irritants and aseptic wounding in the dermis, and *Listeria monocytogenes* peritoneal infection (74). However, unlike infiltrating monocytes (Ly6C^hi^CX3CR1^low^MHC-II^-^), NCMs (Ly6C^low^CX3CR1^hi^MHC-II^+^) assume pro-repair functions. In physiological states, they provide immunosurveillance to endothelial cells and get implicated in repairing leaky vessels upon liver injury (74). Indeed, the vessel-repair function of NCMs has been demonstrated in physiological and pathological conditions involving many organs such as the skin (179, 180). Despite limited investigations of the role of NCMs in response to liver injury, a study on perinatal hepatic NCMs revealed that they overexpress *Il4ra* and *Tgfb1* genes highlighting their anti-inflammatory and pro-repair functions. Authors also demonstrated that hepatic NCMs might be implicated in resistance to rhesus rotavirus-mediated periportal inflammation and suggested these cells as therapeutic targets for diminishing perinatal liver inflammation (181). Indeed, our studies on CCl_4_-induced acute injury showing the influx of NCMs together with monocytes and neutrophils in the necroinflammation phase may reflect implication of NCMs in alleviating inflammation and repairing the ensuing tissue damage (70).

#### 4.3.2 The tissue repair phase of the wound healing response

Tissue repair involves decreased necrotic and inflammatory responses and restoration of structural and functional properties back to homeostasis. Cytokines, chemokines, pro-/anti-inflammatory mediators and growth factors shift the response toward restructuring the tissue, remodeling the ECM, and repopulating the parenchyma at the site of injury. During this phase of the response to acute liver injury, plasma ALT and AST levels decrease concomitantly with a reduction in the area of necrotic tissue (53, 177). An important feature of tissue repair is cell proliferation in the aim of ensuring repopulation of the tissue after injury and necrosis. A fate mapping study demonstrated the capacity of hepatocytes around the portal tracts to divide, proliferate and repopulate the damaged area around central veins resulting from CCl_4_-acute liver injury (182). Similarly, we observed compartmentalized proliferation of tissue cells during the repair phase upon CCl_4_-injury, with hepatocytes proliferating around portal tracts and non-parenchymal cells (including KCs and HSCs) regenerating around injured central veins, underscoring the idea of a spatially-regulated tissue repair after injury (70). Interestingly, such distinctive distribution of cells around the injured area, in this case proliferative cells, is gaining more and more attention and is providing solid evidence of its biological significance.

Efferocytosis, the process of recognition and removal of dead cells by phagocytes, is not only essential for homeostasis, as discussed above, but also important for the resolution of tissue damage following liver injury (183, 184). KCs express Timd4 (57) and MerTK (161, 185), both receptors of the “eat me” signal, phosphatidyl serine (PS) that get exposed on apoptotic cells, underscoring the efferocytic role of these cells during homeostasis and disease conditions. KCs also express scavenger receptors further supporting their phagocytic function (186). The phagocytic and efferocytic functions of liver macrophages become highly relevant during pathogen-induced or toxic injury, due to the need of pathogen clearance, and the removal of dead and aged/senescent cells from the injured tissue, respectively. CLEC4F on KCs mediates the clearance of desialylated platelets induced by bacteria-derived neuraminidases (187). In addition, intravital imaging demonstrated that KCs captured and cleared blood-borne Gram-positive bacteria within minutes of pathogen invasion (188). During liver injury induced by D-galactosamine/LPS, uptake of apoptotic cells by KCs induced a shift toward an anti-inflammatory milieu (TGF-β, IL-4 and IL-10) and alleviated tissue damage (184, 189). The anti-inflammatory pro-resolving phenotype of KCs is also enhanced via the expression of the TAM (Tyro3-Axl-MerTK) family of receptor tyrosine kinases known to be involved in the recognition and removal of apoptotic cells, probably neutrophils (161). Indeed, patients with acute liver failure (ALF) exhibited expansion of MerTK/MHC-II-expressing macrophages, revealed to be KCs with high phagocytic (190) and pro-resolving properties (161), in necrotic areas of the liver. Similarly, MerTK-deficient mice exhibited an aggravated liver injury upon APAP treatment as compared to WT and demonstrated low levels of MHC-II^high^/LyC6^low^ KCs and high infiltration of activated neutrophils (MPO^+^) at the injury site (161). It is noteworthy that KCs in this study were identified as CD45^+^/CD11b^int^/Ly6G^-^/Ly6C^low^/F4/80^hi^/MHC-II^+^. Since none of these markers is KC-specific, it is possible that the KC gate was enriched in macrophages from sources other than the resident pool (e.g., Ly6C^low^ MoMFs) (191). Fittingly, the differentiation of liver-infiltrating monocytes/macrophages into anti-inflammatory pro-resolving MoMFs with high expression of CX3CR1 is enhanced by the release of CX3CL1 from apoptotic cells, further underscoring the importance of apoptosis recognition in damage resolution (71, 184). Moreover, CX3CR1^+^ MoMFs express a wide array of bridge molecules and receptors that mediate the recognition and engulfment of apoptotic cells (177). For instance, PS-recognizing receptor, Stabilin-1 is implicated in the injury-induced switch of inflammatory macrophages (LyC6^hi^) to pro-resolving anti-inflammatory LyC6^low^ macrophages (192). In a model of focal sterile injury, reduced numbers of intrahepatic MoMFs at the injury site in CCR2-deficient mice led to delayed clearance of dead cells (71). Interestingly, in this same model, a subpopulation of GATA-6^+^ peritoneal macrophages infiltrating the liver during the first hours, was implicated in the disassembling of necrotic cells, demonstrating that multiple hepatic macrophage subsets cooperate in the removal of dead cells (50).

##### 4.3.2.1 KCs during repair

During the repair phase, KCs repopulate the injury site originating from the remaining cells or from recruited/differentiated macrophages. This concept will be discussed in more details in subsequent sections. Indeed, a gradual increase in the frequency of KCs is observed from early to late repair (70). Interestingly, studies have shown that during the repair phase of acute toxic injury using APAP and TAA, depleting KCs was associated with delayed repair and an extended period of necrosis and liver dysfunction, underscoring their important role in resolving tissue damage and initiating repair (65–67, 193). A probable underlying mechanism for the tissue protecting function of KCs could be via preventing TNF-α-induced tissue damage as reported in a CCl_4_-injury model where KC depletion triggered infiltration of myeloid cells and upregulation of TNF-α. This process is reversed upon treatment with neutralizing anti-TNF-α Abs (67). As mentioned above, KCs themselves are an important source of TNF-α which contributes to inflammation by inducing the secretion of other pro-inflammatory cytokines and chemokines and promoting the influx of monocyte/macrophages to the injury site (70, 168, 169). Reducing TNF-α decreased hepatic tissue damage in LPS-treated rats (194), however it delayed repair in APAP and partial hepatectomy models via abrogating hepatocyte survival and proliferation processes (167, 169, 195), suggesting a dual role of this cytokine depending on the injury type and the phase of the response.

KCs also contribute to wound healing by producing IL-1β that could exhibit a pro-repair role by inducing matrix metalloproteases (MMPs) expression in HSCs allowing degradation of newly formed ECM (175). Furthermore, IL-1β was shown to trigger VEGF production and regeneration of injured vessels, underscoring its angiogenic signature (174). Another suggested mode of action of tissue-protective KCs is by releasing CCL2 that promotes the influx of inflammatory monocytes that will differentiate into the pro-repair MoMFs implicated in wound healing (53, 66). In addition, in the APAP acute injury model, KCs abrogate the activation of LSECs preventing vessel hyperpermeability and enhancing the expression of hepatic angiogenic genes that promote regeneration of vessels (66, 196). Another tissue repair signature of KCs is assumed via their capacity to restore normal ECM by phagocytizing fibrin, an ECM protein generated upon tissue damage (65, 197).

KCs are also a major source of TGF-β, a pleiotropic cytokine that plays an important role in liver homeostasis and disease conditions (198). TGF-β maintains the liver mass in the physiological state via its growth-inhibiting and pro-apoptotic actions toward hepatocytes but switches to inflammatory, tissue generating and even fibrotic functions during various stages of the liver injury (199, 200). Studies have shown an increased expression of TGF-β in livers under acute and chronic injury conditions (201–203). In addition, this cytokine was shown to regulate plasticity of macrophages and to enhance their transition from pro- to anti-inflammatory cells in the repair phase. Our data in the CCl_4_ acute injury model demonstrated increased expression of TGF-β in the repair phase suggesting its role as a potent inducer of HSC activation and trans-differentiation during this phase (70).

It is noteworthy that an important aspect of KCs and their role in tissue damage resolution is the expression of Trem-2 on their cell surface. Trem-2, a member of the Triggering receptor expressed on myeloid cells (Trem) family, is expressed on cells of the myeloid lineage. In acute and chronic liver injuries, Trem-2 was shown to modulate KCs replenishment from infiltrating monocytes (referred to by the authors as transition macrophages) promoting the shift from pro-inflammatory to repair phase (204). An earlier study demonstrated increased expression of Trem-2 in patients with cirrhotic livers and an association with hepatic injury and inflammatory markers suggesting that Trem-2 functions at counteracting inflammatory events in liver disease (205). The same study, using a *Trem-2^-/-^* mouse model of either acute (APAP) or chronic (CCl_4_) liver injury and bone marrow transplantation experiments underscored the role of Trem-2 as a natural brake on inflammation during various forms of hepatotoxic injury. Authors also demonstrated that the effects of Trem-2 in the context of chronic injury depended on its expression/function in both infiltrating immune cells and resident cells (205).

##### 4.3.2.2 MoMFs during repair

During early repair, MoMFs are the predominant myeloid subpopulation as we demonstrated in the CCl_4_-induced liver injury model (70). This is in line with previous data that highlighted the anti-inflammatory/pro-repair functions of MoMFs in other liver injuries such as APAP- and thermal-induced injuries (53, 71). The pro-repair function of MoMFs was also reported in studies demonstrating increased frequencies of these cells in highly resolving wound areas and an upregulated release of their MMPs (78). Depleting these cells was associated with enhanced fibrosis (78). These studies firmly established that MoMFs at the injury site are generated from recruited inflammatory monocytes where they perform crucial effector functions for the resolution of inflammation and the restoration of tissue homeostasis.

The expression of Arginase-1 (Arg-1) delineates the phenotypic signature of macrophages as pro-repair, in contrast to inflammatory macrophages defined by their iNOS expression, previously denoted as M2 and M1 macrophages, respectively (206, 207). Arg-1 initiates the metabolism of L-arginine, the final products of which, polyamines and proline were shown to enhance cell proliferation and collagen synthesis, respectively in skin injuries. This implies a significant role for Arg-1 in ECM and epithelial remodeling upon injury in the skin (208). In the liver, using the acute CCl_4_ injury model, we observed a temporal association between peak Arg-1 expression in myeloid cells (48 hrs), activation of HSCs and expression of fibrillar collagen (70). This association fits the notion of Arg-1 playing a role in ECM repair, but additional studies are needed to dissect the role of Arg-1 expression in myeloid cells during tissue repair in the liver.

Other factors modulating monocyte differentiation include the macrophage colony-stimulating factor (M-CSF or CSF-1), known to promote differentiation of bone-marrow-derived inflammatory monocytes into MoMFs in peripheral tissues (209–211). In line with this evidence, a CSF1-Fc fusion protein used to treat fibrosis after cessation of TAA-mediated injury was shown to enhance liver growth, to prevent progression and to promote resolution of fibrosis. CSF1-Fc fusion protein also accelerated recovery in a model of hepatectomy (212).

Another important factor is the chemokine CX3CL1, also known as fractalkine. Fractalkine is an adhesion molecule expressed on the surface of activated ECs, smooth muscle cells, skeletal muscle cells, macrophages, neurons, hepatocytes and others (213). It is also released as a chemokine responsible for attracting cells expressing its receptor (CX3CR1) including MoMFs, NCMs, NK cells, and CD8 and γδ T cells (214). Despite its inflammatory role in numerous chronic inflammatory diseases (215), the CX3CL1/CX3CR1 interaction plays a pro-repair function during acute liver injury. CX3CR1 deficiency in mice increased immune cell influx from circulation and enhanced release of pro-inflammatory cytokines such as TNF-α, monocyte chemoattractant protein 1 (MCP1), macrophage inflammatory protein 1β (MIP1β), and regulated upon activation, normal T cell expressed and secreted (RANTES) following CCl_4_ treatment (28). In addition, infiltrating MoMFs lacking CX3CR1 exhibited higher apoptotic activity than control CX3CR1^+^ cells (176). The pro-repair function of the CX3CL1/CX3CR1 pair was further demonstrated via its implication in the phenotypic switch of pro-inflammatory (TNF-α-, iNOS-producers) into anti-inflammatory/pro-repair (IL-10- and Arg-1-producers) macrophages (28, 176). In line with a potential role of CX3CL1 in tissue repair upon CCl_4_ acute liver injury, we observed that peak hepatic upregulation of CX3CL1/CX3CR1 was temporally associated with decreased inflammatory and tissue damage markers, and increased proliferation of tissue cells and ECM synthesis (70). Further investigations are needed to better define the role of the immune mediators mentioned above in the various stages of liver-injury response.

##### 4.3.2.3 Neutrophils during repair

The other major myeloid cell population implicated tissue repair upon liver injury is neutrophils. While contributing to inflammation and tissue necrosis via their numerous effector functions, neutrophils also undertake pro-resolving functions in various injury settings. Indeed, by promoting angiogenesis and re-epithelization, neutrophils were shown to be critical for tissue repair in models of skin injury (216). Similarly, neutrophils are involved in the break-up of damaged vessels, the assembly of new ones and the remodeling of ECM upon focal thermal sterile injury of the liver, enabling tissue damage resolution (149). Interestingly, the phagocytic function of neutrophils contribute to tissue repair, by phagocytosing dead cells and inflammatory debris from the necrotic tissue (44, 149, 217). Additional pro-resolving activities of neutrophils are exhibited via their release of ROS which was shown to enhance the differentiation of the pro-inflammatory monocytes/macrophages (Ly6C^hi^CX3CR1^-^) into pro-resolving (Ly6C^lo^CX3CR1^+^) MoMFs (44). Interestingly, in the CCl_4_ acute liver injury model, we observed two waves of neutrophil infiltration: one in necroinflammation and the other peaking during the repair phase. Since each wave is observed in a different phase of the response, and given the distinctive immune signature of each phase, it is tempting to speculate that neutrophil subsets with different functions are involved at each wave (70). Indeed, several studies have described distinct neutrophil subsets at the transcriptional, functional, and phenotypic level during homeostasis and disease (reviewed in (218, 219)). Further studies examining the different functional subsets of neutrophils during acute and chronic liver injury are warranted.

#### 4.3.3 Spatial and temporal distribution of hepatic macrophages during injury

KCs zonation along the porto-central axis strongly influences their morphology and function. KCs in the periportal zone are of higher density and larger size, with a more pronounced phagocytic activity and milder responsiveness to inflammatory stimuli as compared to those in the centrilobular zone (220–222). Such zonation is established at the time of weaning in mice and is dependent on the gut microbiota. Indeed, enrichment of KCs in the periportal area was established at the time of weaning in specific pathogen-free (SPF) mice but was absent in the livers of germ-free (GF) mice (68). Treatment of GF mice with LPS, or co-housing with SPF mice, induced KC accumulation in the periportal zone. Conversely, antibiotic treatment of SPF mice, eliminated KC polarization (68). A crosstalk with LSECs expressing a gradient of the chemokine CXCL9 affects the KC asymmetrical localization along the porto-central axis (68). KC zonation was shown to be critical for preventing the spread of the bacterium *Listeria monocytogenes* to the circulation and thereafter to the spleen, further outlining the importance of the uneven distribution of KCs along the sinusoidal axis (68) in maintaining liver homeostasis and immune defense. The spatial distribution of KCs and MoMFs during acute liver injury is an active area of research. It is noteworthy that most studies on acute liver injury did not use specific identification markers of the different macrophage subpopulations like MoMFs, peritoneal macrophages, liver capsular macrophages and resident KCs (53, 177), thus precluding appropriate description of the localization of these cells at the injury site.

Our group and others described the spatial and temporal distribution of hepatic macrophages in acute liver injury models. During the early repair phase of the response to APAP-induced liver injury, MoMFs were the dominant hepatic myeloid cells and were concentrated in necrotic areas around central veins through the interaction of their surface receptor CCR2 with its ligand CCL2 (53, 177). During this stage, a pro-resolving role has been proposed to MoMFs thus alleviating necrosis and reducing tissue damage. At later stages of the repair phase (120 hrs post-injury), MoMFs were shown to be scarcely present in the liver (53). In the focal thermal liver injury model, inflammatory monocytes identified as Ly6C^hi^CX3CR1^-^ cells, rapidly translocate into the liver and more specifically around the injury site in a CCR2-dependent mechanism. With the progression of injury, i.e. at 24-48 hrs post-thermal probe insult, the recruited monocytes differentiate into MoMFs, then transmigrate deep in the necrotic area in an IL-4- and IL-10-dependent manner and efficiently contribute to dead cell clearance and ECM remodeling (71). In the same line of evidence, we reported similar findings with respect to MoMFs location and role in the necroinflammatory and early repair stages of the response to acute CCl_4_-induced injury. Our study further described a distinctive interaction of MoMFs with other immune cells as well as parenchymal cells in the affected area (70).

**Figure 2:**
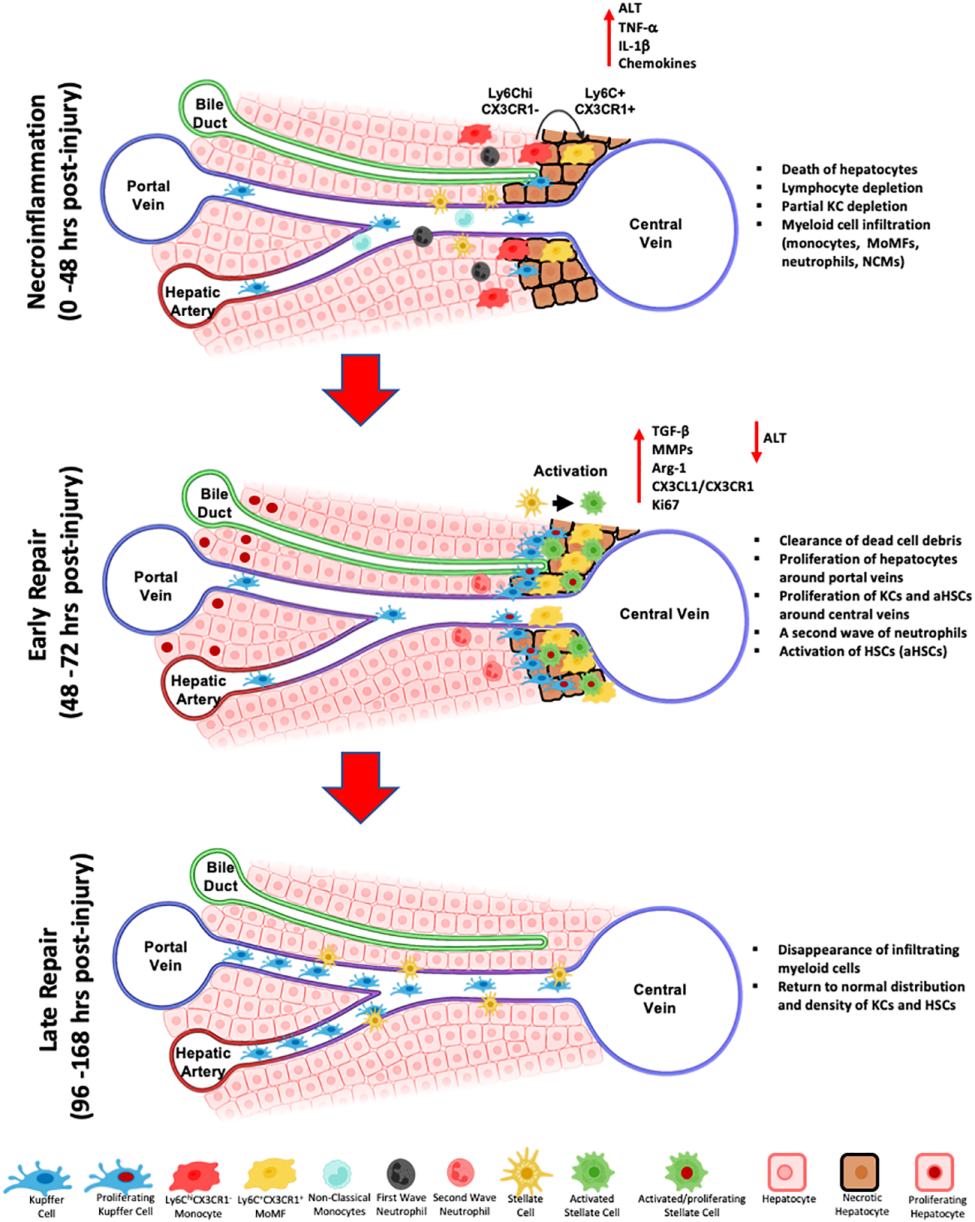
Schematic representation of the wound healing response during toxin-induced acute liver injury. The first response phase termed necroinflammation, is characterized by the death of pericentral hepatocytes, a partial depletion of hepatic lymphocytes, decrease of Kupffer cells, the influx of myeloid cells, namely Ly6C^hi^CX3CR1^-^ inflammatory monocytes, differentiated Ly6C^+^CX3CR1^+^ MoMFs, non-classical monocytes (NCMs), and a first wave of neutrophils, and the release of pro-inflammatory cytokines and chemokines, including IL-1β and TNF-α. Subsequently, during the early repair, MoMFs further differentiate and proliferate, they relocate to the inner necrotic regions around central veins and participate to the activation of HSCs and the clearing of dead hepatocytes. KCs proliferate from remaining KCs and repopulate the outermost regions of the injury around central veins. A second wave of infiltrating neutrophils contribute to injury repair by clearing dead cell debris and release of ROS. During late repair, MoMFs and neutrophils are depleted, and the cluster of KCs and HSCs around central veins dissipate to re-establish the homeostatic spatial distribution of resident cell subsets.

As for KCs, they are partially depleted during necroinflammation in the APAP-induced liver injury model and repopulate the injury site through proliferation of the remaining cells during the resolution/repair phase (53). We further confirmed these observations in the CCl_4_-acute injury. Indeed, using multiplex IF, we demonstrated that during necroinflammation, KCs that escaped death formed ring-like structures at the periphery of the injury site that is filled with MoMFs. With the progression to early repair, KCs relocated closer to the injured area where they proliferated and formed dense aggregates that disappeared at the end stage of repair to re-establish the homeostatic distribution of KCs (70). **Figure 2** summarizes the various phases of the response to acute liver injury as described above. It is noteworthy, that our observations of KCs relocation, suggesting a motile phenotype capable of relocating during the response to acute liver injury, contradict the previous notion of KCs as sessile cells (47, 87, 223). In this context, KCs, were shown to exhibit a fixed position during several hours (87), or even under inflammatory stimuli from influenza-induced CD8 T cells (47). However, our observations of KCs relocation during necroinflammation and repair could be explained by a migratory profile of KCs (70), at least during CCl_4_ acute liver injuries. It can be also explained by selective and localized depletion/proliferation/contraction cycles. Altogether, there is a need for better assessment of the spatial behavior of KCs in various models of hepatic inflammation using newer technologies and reporter systems. Finally, results demonstrating the depletion of KCs during the early inflammatory phase (24 h post-injury) and the subsequent infiltration of MoMFs into the site of injury, are consistent with the idea that activation of KCs and their death thereafter promote the influx of circulatory monocytes and their differentiation into MoMFs (70, 224, 225). In acetaminophen-induced ALF patients, the hepatic necrosis at the centrilobular level is associated with an enhanced macrophage response at the injury site. In this context, resident KCs exhibit proliferative phenotype and inflammatory monocytes get recruited in a CCR2-dependent manner. Both macrophage populations will ensure an anti-inflammatory/repair milieu for a proper resolution of tissue damage (185, 226).

#### 4.3.4 Origin of the replenished KC pool

While their partial or complete depletion is characteristically seen in several injury models, KCs repopulating the liver during repair can be derived from different origins depending on the model (46, 52, 53, 92). In the naïve liver, the KC pool is exclusively repopulated through self-renewal (84). However, under pathological states, KCs might also originate from infiltrating monocytes/MoMFs that differentiate into what is termed, monocyte-derived KCs (MoKCs) or bone-marrow-derived KCs (BM-KCs) (52, 53, 179, 227). What determines the origin of repopulating KCs is not clear. In APAP-injured liver, the KC pool was replenished from pre-existing KCs. In fact, in this model, KCs and infiltrating MoMFs exhibited transcriptomically distinct profiles revealed by the differential expression of a wide range of wound healing genes (e.g., scavenger receptors, C-type lectins, complement receptors and ECM-remodeling enzymes) and highlighting the distinctive function of each of these macrophage subtypes (53). On the other hand, and in other models of liver injury, e.g., the radiation- or diphtheria toxin- induced injuries, hepatic MoMFs were capable of differentiating into MoKCs and repopulating the KC pool after injury-induced death of KCs. MoKCs expressed transcription factors and canonical markers of KCs, e.g., CLEC4F. They also assumed the spatial location of KCs in the sinusoidal space and their stellar morphology. MoKCs were also capable to self-maintain by proliferating similarly to the original embryonically-derived KCs (19, 46, 52). The newly differentiated MoKCs and the original KCs undertook similar as well as different functions in the physiological context or response to injury. They exhibited comparable phagocytic activity against effete red blood cells and similar response to Leishmania (46). In contrast, MoKCs were better phagocytes of bacteria like *Listeria monocytogenes* and *Neisseria meningitides* than embryonic KCs (46), and more inflammatory in mouse models of NASH (163).

#### 4.3.5 Interaction of hepatic macrophages with HSCs

Hepatic macrophages communicate with numerous cell types in their environment during homeostasis and more importantly under pathological conditions, by either direct physical contact, e.g., the interaction of KCs with CD8 T cells (228), or indirectly via the release of various immune mediators as discussed in the previous sections. One of the most interesting intercellular interactions in the hepatic tissue are between hepatic macrophages and HSCs. Bonnardel et al. showed that KCs and HSCs are in direct contact in a one-to-one ratio in the normal liver through fenestrations present in the endothelial walls of the sinusoids (19). During the wound healing response, activation of HSCs is a characteristic feature of the repair and fibrotic processes in acute and chronic liver injuries, respectively (229, 230). By undergoing myofibroblastic transformation, aHSCs express high levels of alpha smooth muscle actin (αSMA) and ECM proteins including collagen (231). In this context, both KCs and MoMFs were shown to contribute to the activation of HSCs. KCs activate HSCs by producing various pro-inflammatory mediators and growth factors (232, 233). They release the platelet derived growth factor (PDGF) and stimulate the upregulation of its receptor on HSCs, inducing their proliferation and activation (234, 235). In addition, TGF-β produced by activated KCs during liver injury in a MerTK-ERK1/2-dependent signaling, promotes the activation of HSCs and collagen deposition (233). Furthermore, in an *in vitro* model mimicking the *in vivo* intercellular communications, KCs released reactive oxygen species and IL-6 that enhanced the synthesis of collagen and reduced its turnover from co-cultured HSCs by upregulating *Col1a1* and *Col1a2* gene expression and reducing MMP13 activity, respectively (236). In the CCl_4_ acute injury model, we detected KCs in direct contact with quiescent HSCs in the steady state, and to some degree with aHSCs at all-time points of the wound healing response. However, as the response progressed, the colocalization and interaction of aHSCs with MoMFs became more evident than with KCs. This suggests that in the CCl_4_ acute liver injury condition, resident macrophages act as first line activators of HSCs, and that this function is taken over by MoMFs at later stages of the response (70).

We have demonstrated colocalization and a direct contact between αSMA^+^ aHSCs and MoMFs at the early repair phase of the CCl_4_-induced injury response (70). These MoMFs highly expressed TNF-α and IL-13, important activators of HSC, at early repair and temporally associated with a fully activated phenotype of HSCs at this stage (70). A similar interplay between MoMFs and their neighboring HSCs was also demonstrated in liver fibrosis. MoMFs in fibrotic livers were shown to exhibit high gene expression of factors associated with fibrosis (e.g., *Vegfa* or *Igf1*), with HSC survival (e.g., *Il1b* and *Tnfa*), and with ECM modulation (237). Altogether, these studies confirm the physical and functional communication between hepatic macrophages and HSCs and its implication in the response to injury.

## 5 Concluding remarks and future directions

In summary, the immune response to liver injury is a complex yet coordinated process involving numerous cells with individual roles as well as interrelated functions. Liver macrophages are key players in the response to acute liver injury. The wound healing response is initiated by a necroinflammatory phase characterized by death of hepatic cells, partial depletion of resident lymphocytes and KCs, infiltration of myeloid cells into the injury site and increased release of pro-inflammatory mediators. Subsequently, in early repair, more infiltrating monocytes differentiate into MoMFs that further proliferate and dominate the inner necrotic area around central veins, phagocytize dead hepatocytes and activate HSCs. At late repair, the re-establishment of the homeostatic distribution of resident cell subsets takes place **(** **Figure 2** **)**. Nevertheless, it is important to note that deviations from the proposed mechanisms could take place in different models of injury and at different conditions (e.g., different doses) and that further investigations of these effects are needed.

The technological advances allowing better understanding of the hepatic immune signatures in the context of liver disease, will pave the way for a more efficient translation of findings from animal models to humans, the identification of novel targets for disease management and the development of efficient therapeutic molecules or approaches. Hepatic macrophages are major players at different levels of liver injury with a multifaceted inflammatory, pro-fibrotic and pro-resolving role and thus contribute to tissue damage, repair, regeneration, and fibrosis. As such, macrophages constitute an attractive target for a better understanding of liver pathologies and the development of therapeutic or interventional treatment approaches for liver disease.

## Author contributions

GSH wrote the first draft of the manuscript. MFM performed the initial review of the literature. NHS, the corresponding author, oversaw the overall process and revised different versions of the manuscript. All authors reviewed the article and approved the final version.

## References

[1] Racanelli V Rehermann B. The liver as an immunological organ. Hepatology (2006) 43(2 Suppl 1):S54–62. doi: 10.1002/hep.21060 16447271

[2] Hernandez-Gea V Friedman SL. Pathogenesis of liver fibrosis. Annu Rev Pathol (2011) 6:425–56. doi: 10.1146/annurev-pathol-011110-130246 21073339

[3] Kietzmann T. Metabolic zonation of the liver: The oxygen gradient revisited. Redox Biol (2017) 11:622–30. doi: 10.1016/j.redox.2017.01.012 PMC5257182 28126520

[4] Shetty S Lalor PF Adams DH. Liver sinusoidal endothelial cells - gatekeepers of hepatic immunity. Nat Rev Gastroenterol Hepatol (2018) 15(9):555–67. doi: 10.1038/s41575-018-0020-y PMC7096836 29844586

[5] Stan RV Tse D Deharvengt SJ Smits NC Xu Y Luciano MR. The diaphragms of fenestrated endothelia: Gatekeepers of vascular permeability and blood composition. Dev Cell (2012) 23(6):1203–18. doi: 10.1016/j.devcel.2012.11.003 PMC3525343 23237953

[6] Braet F Wisse E. Structural and functional aspects of liver sinusoidal endothelial cell fenestrae: A review. Comp Hepatol (2002) 1(1):1. doi: 10.1186/1476-5926-1-1 12437787 PMC131011

[7] Løvdal T Andersen E Brech A Berg T. Fc receptor mediated endocytosis of small soluble immunoglobulin G immune complexes in Kupffer and endothelial cells from rat liver. J Cell Sci (2000) 113(Pt 18):3255–66. doi: 10.1242/jcs.113.18.3255 10954423

[8] Wilkinson AL Qurashi M Shetty S. The role of sinusoidal endothelial cells in the axis of inflammation and cancer within the liver. Front Physiol (2020) 11:990. doi: 10.3389/fphys.2020.00990 32982772 PMC7485256

[9] Li R Oteiza A Sørensen KK McCourt P Olsen R Smedsrød B. Role of liver sinusoidal endothelial cells and stabilins in elimination of oxidized low-density lipoproteins. Am J Physiol Gastrointest Liver Physiol (2011) 300(1):G71–81. doi: 10.1152/ajpgi.00215.2010 PMC3025507 21030611

[10] Mates JM Yao Z Cheplowitz AM Suer O Phillips GS Kwiek JJ. Mouse Liver Sinusoidal Endothelium Eliminates HIV-Like Particles from Blood at a Rate of 100 Million per Minute by a Second-Order Kinetic Process. Front Immunol (2017) 8:35. doi: 10.3389/fimmu.2017.00035 28167948 PMC5256111

[11] Huang S Wu J Gao X Zou S Chen L Yang X. LSECs express functional NOD1 receptors: A role for NOD1 in LSEC maturation-induced T cell immunity *in vitro* . Mol Immunol (2018) 101:167–75. doi: 10.1016/j.molimm.2018.06.002 29944986

[12] Martin-Armas M Simon-Santamaria J Pettersen I Moens U Smedsrød B Sveinbjørnsson B. Toll-like receptor 9 (TLR9) is present in murine liver sinusoidal endothelial cells (LSECs) and mediates the effect of CpG-oligonucleotides. J Hepatol (2006) 44(5):939–46. doi: 10.1016/j.jhep.2005.09.020 16458386

[13] Wu J Meng Z Jiang M Zhang E Trippler M Broering R. Toll-like receptor-induced innate immune responses in non-parenchymal liver cells are cell type-specific. Immunology (2010) 129(3):363–74. doi: 10.1111/j.1365-2567.2009.03179.x PMC2826681 19922426

[14] Böttcher JP Schanz O Garbers C Zaremba A Hegenbarth S Kurts C. IL-6 trans-signaling-dependent rapid development of cytotoxic CD8+ T cell function. Cell Rep (2014) 8(5):1318–27. doi: 10.1016/j.celrep.2014.07.008 25199826

[15] Knolle PA Limmer A. Neighborhood politics: the immunoregulatory function of organ-resident liver endothelial cells. Trends Immunol (2001) 22(8):432–7. doi: 10.1016/S1471-4906(01)01957-3 11473832

[16] Knolle PA Uhrig A Hegenbarth S Löser E Schmitt E Gerken G. IL-10 down-regulates T cell activation by antigen-presenting liver sinusoidal endothelial cells through decreased antigen uptake via the mannose receptor and lowered surface expression of accessory molecules. Clin Exp Immunol (1998) 114(3):427–33. doi: 10.1046/j.1365-2249.1998.00713.x PMC1905120 9844054

[17] Knolle PA Wohlleber D. Immunological functions of liver sinusoidal endothelial cells. Cell Mol Immunol (2016) 13(3):347–53. doi: 10.1038/cmi.2016.5 PMC4856811 27041636

[18] Schurich A Berg M Stabenow D Böttcher J Kern M Schild HJ. Dynamic regulation of CD8 T cell tolerance induction by liver sinusoidal endothelial cells. J Immunol (2010) 184(8):4107–14. doi: 10.4049/jimmunol.0902580 20212092

[19] Bonnardel J T'Jonck W Gaublomme D Browaeys R Scott CL Martens L. Stellate cells, hepatocytes, and endothelial cells imprint the kupffer cell identity on monocytes colonizing the liver macrophage niche. Immunity (2019) 51(4):638–54.e9. doi: 10.1016/j.immuni.2019.08.017 31561945 PMC6876284

[20] Geerts A. History, heterogeneity, developmental biology, and functions of quiescent hepatic stellate cells. Semin Liver Dis (2001) 21(3):311–35. doi: 10.1055/s-2001-17550 11586463

[21] Bachem MG Melchior R Gressner AM. The role of thrombocytes in liver fibrogenesis: effects of platelet lysate and thrombocyte-derived growth factors on the mitogenic activity and glycosaminoglycan synthesis of cultured rat liver fat storing cells. J Clin Chem Clin Biochem (1989) 27(9):555–65. doi: 10.1515/cclm.1989.27.9.555 2607320

[22] Canbay A Taimr P Torok N Higuchi H Friedman S Gores GJ. Apoptotic body engulfment by a human stellate cell line is profibrogenic. Lab Invest (2003) 83(5):655–63. doi: 10.1097/01.LAB.0000069036.63405.5C 12746475

[23] Friedman SL. Hepatic stellate cells: protean, multifunctional, and enigmatic cells of the liver. Physiol Rev (2008) 88(1):125–72. doi: 10.1152/physrev.00013.2007 PMC2888531 18195085

[24] Jarnagin WR Rockey DC Koteliansky VE Wang SS Bissell DM. Expression of variant fibronectins in wound healing: cellular source and biological activity of the EIIIA segment in rat hepatic fibrogenesis. J Cell Biol (1994) 127(6 Pt 2):2037–48. doi: 10.1083/jcb.127.6.2037 PMC2120289 7806580

[25] Novo E Marra F Zamara E Valfrè di Bonzo L Caligiuri A Cannito S. Dose dependent and divergent effects of superoxide anion on cell death, proliferation, and migration of activated human hepatic stellate cells. Gut (2006) 55(1):90–7. doi: 10.1136/gut.2005.069633 PMC1856394 16041064

[26] Mederacke I Hsu CC Troeger JS Huebener P Mu X Dapito DH. Fate tracing reveals hepatic stellate cells as dominant contributors to liver fibrosis independent of its aetiology. Nat Commun (2013) 4:2823. doi: 10.1038/ncomms3823 24264436 PMC4059406

[27] Tsuchida T Friedman SL. Mechanisms of hepatic stellate cell activation. Nat Rev Gastroenterol Hepatol (2017) 14(7):397–411. doi: 10.1038/nrgastro.2017.38 28487545

[28] Aoyama T Inokuchi S Brenner DA Seki E. CX3CL1-CX3CR1 interaction prevents carbon tetrachloride-induced liver inflammation and fibrosis in mice. Hepatology (2010) 52(4):1390–400. doi: 10.1002/hep.23795 PMC2947579 20683935

[29] Baiocchini A Del Nonno F Taibi C Visco-COmandini U D'Offizi G Piacentini M. Liver sinusoidal endothelial cells (LSECs) modifications in patients with chronic hepatitis C. Sci Rep (2019) 9(1):8760. doi: 10.1038/s41598-019-45114-1 31217430 PMC6584733

[30] Bourd-Boittin K Basset L Bonnier D L'Helgoualc'h A Samson M Théret N. CX3CL1/fractalkine shedding by human hepatic stellate cells: contribution to chronic inflammation in the liver. J Cell Mol Med (2009) 13(8a):1526–35. doi: 10.1111/j.1582-4934.2009.00787.x PMC3828864 19432809

[31] Schwabe RF Bataller R Brenner DA. Human hepatic stellate cells express CCR5 and RANTES to induce proliferation and migration. Am J Physiol Gastrointest Liver Physiol (2003) 285(5):G949–58. doi: 10.1152/ajpgi.00215.2003 12829440

[32] Thompson KC Trowern A Fowell A Marathe M Haycock C Arthur MJ. Primary rat and mouse hepatic stellate cells express the macrophage inhibitor cytokine interleukin-10 during the course of activation *In vitro* . Hepatology (1998) 28(6):1518–24. doi: 10.1002/hep.510280611 9828215

[33] Tiggelman AM Boers W Linthorst C Brand HS Sala M Chamuleau RA. Interleukin-6 production by human liver (myo)fibroblasts in culture. Evidence for a regulatory role of LPS, IL-1 beta and TNF alpha. J Hepatol (1995) 23(3):295–306. doi: 10.1016/S0168-8278(95)80009-3 8550994

[34] Wang SC Ohata M Schrum L Rippe RA Tsukamoto H. Expression of interleukin-10 by in *vitro* and in *vivo* activated hepatic stellate cells. J Biol Chem (1998) 273(1):302–8. doi: 10.1074/jbc.273.1.302 9417080

[35] Marra F Tacke F. Roles for chemokines in liver disease. Gastroenterology (2014) 147(3):577–94.e1. doi: 10.1053/j.gastro.2014.06.043 25066692

[36] Getachew A Abbas N You K Yang Z Hussain M Huang X. SAA1/TLR2 axis directs chemotactic migration of hepatic stellate cells responding to injury. iScience (2021) 24(5):102483. doi: 10.1016/j.isci.2021.102483 34113824 PMC8169952

[37] Passino MA Adams RA Sikorski SL Akassoglou K. Regulation of hepatic stellate cell differentiation by the neurotrophin receptor p75NTR. Science (2007) 315(5820):1853–6. doi: 10.1126/science.1137603 17395831

[38] Doeing DC Borowicz JL Crockett ET. Gender dimorphism in differential peripheral blood leukocyte counts in mice using cardiac, tail, foot, and saphenous vein puncture methods. BMC Clin Pathol (2003) 3(1):3. doi: 10.1186/1472-6890-3-3 12971830 PMC201031

[39] Mestas J Hughes CC. Of mice and not men: differences between mouse and human immunology. J Immunol (2004) 172(5):2731–8. doi: 10.4049/jimmunol.172.5.2731 14978070

[40] Rosales C. Neutrophils at the crossroads of innate and adaptive immunity. J Leukoc Biol (2020) 108(1):377–96. doi: 10.1002/JLB.4MIR0220-574RR 32202340

[41] Segal AW. How neutrophils kill microbes. Annu Rev Immunol (2005) 23:197–223. doi: 10.1146/annurev.immunol.23.021704.115653 15771570 PMC2092448

[42] Liu K Wang FS Xu R. Neutrophils in liver diseases: pathogenesis and therapeutic targets. Cell Mol Immunol (2021) 18(1):38–44. doi: 10.1038/s41423-020-00560-0 33159158 PMC7852892

[43] Ramaiah SK Jaeschke H. Role of neutrophils in the pathogenesis of acute inflammatory liver injury. Toxicol Pathol (2007) 35(6):757–66. doi: 10.1080/01926230701584163 17943649

[44] Yang W Tao Y Wu Y Zhao X Ye W Zhao D. Neutrophils promote the development of reparative macrophages mediated by ROS to orchestrate liver repair. Nat Commun (2019) 10(1):1076. doi: 10.1038/s41467-019-09046-8 30842418 PMC6403250

[45] Guillot A Tacke F. Liver macrophages: Old dogmas and new insights. Hepatol Commun (2019) 3(6):730–43. doi: 10.1002/hep4.1356 PMC6545867 31168508

[46] Beattie L Sawtell A Mann J Frame TCM Teal B de Labastida Rivera F. Bone marrow-derived and resident liver macrophages display unique transcriptomic signatures but similar biological functions. J Hepatol (2016) 65(4):758–68. doi: 10.1016/j.jhep.2016.05.037 PMC5028381 27262757

[47] Klein I Cornejo JC Polakos NK John B Wuensch SA Topham DJ. Kupffer cell heterogeneity: functional properties of bone marrow derived and sessile hepatic macrophages. Blood (2007) 110(12):4077–85. doi: 10.1182/blood-2007-02-073841 PMC2190614 17690256

[48] MacParland SA Liu JC Ma XZ Innes BT Bartczak AM Gage BK. Single cell RNA sequencing of human liver reveals distinct intrahepatic macrophage populations. Nat Commun (2018) 9(1):4383. doi: 10.1038/s41467-018-06318-7 30348985 PMC6197289

[49] Sierro F Evrard M Rizzetto S Melino M Mitchell AJ Florido M. A liver capsular network of monocyte-derived macrophages restricts hepatic dissemination of intraperitoneal bacteria by neutrophil recruitment. Immunity (2017) 47(2):374–88.e6. doi: 10.1016/j.immuni.2017.07.018 28813662

[50] Wang J Kubes P. A reservoir of mature cavity macrophages that can rapidly invade visceral organs to affect tissue repair. Cell (2016) 165(3):668–78. doi: 10.1016/j.cell.2016.03.009 27062926

[51] Jayakumar P Laganson A Deng M. GATA6(+) peritoneal resident macrophage: The immune custodian in the peritoneal cavity. Front Pharmacol (2022) 13:866993. doi: 10.3389/fphar.2022.866993 35401237 PMC8984154

[52] Scott CL Zheng F De Baetselier P Martens L Saeys Y De Prijck S. Bone marrow-derived monocytes give rise to self-renewing and fully differentiated Kupffer cells. Nat Commun (2016) 7:10321. doi: 10.1038/ncomms10321 26813785 PMC4737801

[53] Zigmond E Samia-Grinberg S Pasmanik-Chor M Brazowski E Shibolet O Halpern Z. Infiltrating monocyte-derived macrophages and resident kupffer cells display different ontogeny and functions in acute liver injury. J Immunol (2014) 193(1):344–53. doi: 10.4049/jimmunol.1400574 24890723

[54] Li W Chang N Li L. Heterogeneity and function of kupffer cells in liver injury. Front Immunol (2022) 13:940867. doi: 10.3389/fimmu.2022.940867 35833135 PMC9271789

[55] Patel A van de Poll MC Greve JW Buurman WA Fearon KC McNally SJ. Early stress protein gene expression in a human model of ischemic preconditioning. Transplantation (2004) 78(10):1479–87. doi: 10.1097/01.TP.0000144182.27897.1E 15599312

[56] Scott CL Guilliams M. The role of Kupffer cells in hepatic iron and lipid metabolism. J Hepatol (2018) 69(5):1197–9. doi: 10.1016/j.jhep.2018.02.013 PMC7611037 30001821

[57] Theurl I Hilgendorf I Nairz M Tymoszuk P Haschka D Asshoff M. On-demand erythrocyte disposal and iron recycling requires transient macrophages in the liver. Nat Med (2016) 22(8):945–51. doi: 10.1038/nm.4146 PMC4957133 27428900

[58] Wang Y van der Tuin S Tjeerdema N van Dam AD Rensen SS Hendrikx T. Plasma cholesteryl ester transfer protein is predominantly derived from Kupffer cells. Hepatology (2015) 62(6):1710–22. doi: 10.1002/hep.27985 26174697

[59] Deppermann C Kratofil RM Peiseler M David BA Zindel J Castanheira F. Macrophage galactose lectin is critical for Kupffer cells to clear aged platelets. J Exp Med (2020) 217(4):e20190723. doi: 10.1084/jem.20190723 31978220 PMC7144524

[60] Terpstra V van Berkel TJ. Scavenger receptors on liver Kupffer cells mediate the in *vivo* uptake of oxidatively damaged red blood cells in mice. Blood (2000) 95(6):2157–63. doi: 10.1182/blood.V95.6.2157 10706889

[61] Guilliams M Bonnardel J Haest B Vanderborght B Wagner C Remmerie A. Spatial proteogenomics reveals distinct and evolutionarily conserved hepatic macrophage niches. Cell (2022) 185(2):379–96.e38. doi: 10.1016/j.cell.2021.12.018 35021063 PMC8809252

[62] Heymann F Peusquens J Ludwig-Portugall I Kohlhepp M Ergen C Niemietz P. Liver inflammation abrogates immunological tolerance induced by Kupffer cells. Hepatology (2015) 62(1):279–91. doi: 10.1002/hep.27793 25810240

[63] Tay SS Wong YC McDonald DM Wood NA Roediger B Sierro F. Antigen expression level threshold tunes the fate of CD8 T cells during primary hepatic immune responses. Proc Natl Acad Sci USA (2014) 111(25):E2540–9. doi: 10.1073/pnas.1406674111 PMC4078818 24927525

[64] McDonald B Pittman K Menezes GB Hirota SA Slaba I Waterhouse CC. Intravascular danger signals guide neutrophils to sites of sterile inflammation. Science (2010) 330(6002):362–6. doi: 10.1126/science.1195491 20947763

[65] Golbar HM Izawa T Wijesundera KK Bondoc A Tennakoon AH Kuwamura M. Depletion of hepatic macrophages aggravates liver lesions induced in rats by thioacetamide (TAA). Toxicol Pathol (2016) 44(2):246–58. doi: 10.1177/0192623315621191 26957569

[66] Holt MP Yin H Ju C. Exacerbation of acetaminophen-induced disturbances of liver sinusoidal endothelial cells in the absence of Kupffer cells in mice. Toxicol Lett (2010) 194(1-2):34–41. doi: 10.1016/j.toxlet.2010.01.020 20123118

[67] Sato A Nakashima H Nakashima M Ikarashi M Nishiyama K Kinoshita M. Involvement of the TNF and FasL produced by CD11b Kupffer cells/macrophages in CCl4-induced acute hepatic injury. PLoS One (2014) 9(3):e92515. doi: 10.1371/journal.pone.0092515 24667392 PMC3965417

[68] Gola A Dorrington MG Speranza E Sala C Shih RM Radtke AJ. Commensal-driven immune zonation of the liver promotes host defence. Nature (2021) 589(7840):131–6. doi: 10.1038/s41586-020-2977-2 PMC8691525 33239787

[69] Wong CH Jenne CN Petri B Chrobok NL Kubes P. Nucleation of platelets with blood-borne pathogens on Kupffer cells precedes other innate immunity and contributes to bacterial clearance. Nat Immunol (2013) 14(8):785–92. doi: 10.1038/ni.2631 PMC4972575 23770641

[70] Flores Molina M Abdelnabi MN Mazouz S Villafranca-Baughman D Trinh VQ Muhammad S. Distinct spatial distribution and roles of Kupffer cells and monocyte-derived macrophages in mouse acute liver injury. Front Immunol (2022) 13:994480. doi: 10.3389/fimmu.2022.994480 36248843 PMC9562324

[71] Dal-Secco D Wang J Zeng Z Kolaczkowska E Wong CH Petri B. A dynamic spectrum of monocytes arising from the in *situ* reprogramming of CCR2+ monocytes at a site of sterile injury. J Exp Med (2015) 212(4):447–56. doi: 10.1084/jem.20141539 PMC4387291 25800956

[72] Zeiner PS Preusse C Blank AE Zachskorn C Baumgarten P Caspary L. MIF receptor CD74 is restricted to microglia/macrophages, associated with a M1-polarized immune milieu and prolonged patient survival in gliomas. Brain Pathol (2015) 25(4):491–504. doi: 10.1111/bpa.12194 25175718 PMC8029437

[73] Blériot C Ginhoux F. Understanding the heterogeneity of resident liver macrophages. Front Immunol (2019) 10:2694. doi: 10.3389/fimmu.2019.02694 31803196 PMC6877662

[74] Auffray C Fogg D Garfa M Elain G Join-Lambert O Kayal S. Monitoring of blood vessels and tissues by a population of monocytes with patrolling behavior. Science (2007) 317(5838):666–70. doi: 10.1126/science.1142883 17673663

[75] Tahir S Steffens S. Nonclassical monocytes in cardiovascular physiology and disease. Am J Physiol Cell Physiol (2021) 320(5):C761–C70. doi: 10.1152/ajpcell.00326.2020 33596150

[76] Thomas GD Hamers AAJ Nakao C Marcovecchio P Taylor AM McSkimming C. Human blood monocyte subsets: A new gating strategy defined using cell surface markers identified by mass cytometry. Arterioscler Thromb Vasc Biol (2017) 37(8):1548–58. doi: 10.1161/ATVBAHA.117.309145 PMC5828170 28596372

[77] Grun JL Manjarrez-Reyna AN Gomez-Arauz AY Leon-Cabrera S Ruckert F Fragoso JM. High-density lipoprotein reduction differentially modulates to classical and nonclassical monocyte subpopulations in metabolic syndrome patients and in LPS-stimulated primary human monocytes *in vitro* . J Immunol Res (2018) 2018:2737040. doi: 10.1155/2018/2737040 29850624 PMC5903324

[78] Ramachandran P Pellicoro A Vernon MA Boulter L Aucott RL Ali A. Differential Ly-6C expression identifies the recruited macrophage phenotype, which orchestrates the regression of murine liver fibrosis. Proc Natl Acad Sci U S A (2012) 109(46):E3186–95. doi: 10.1073/pnas.1119964109 PMC3503234 23100531

[79] Fabre T Barron AMS Christensen SM Asano S Bound K Lech MP. Identification of a broadly fibrogenic macrophage subset induced by type 3 inflammation. Sci Immunol (2023) 8(82):eadd8945. doi: 10.1126/sciimmunol.add8945 37027478

[80] Wynn TA Vannella KM. Macrophages in tissue repair, regeneration, and fibrosis. Immunity (2016) 44(3):450–62. doi: 10.1016/j.immuni.2016.02.015 PMC4794754 26982353

[81] Gomez Perdiguero E Klapproth K Schulz C Busch K Azzoni E Crozet L. Tissue-resident macrophages originate from yolk-sac-derived erythro-myeloid progenitors. Nature (2015) 518(7540):547–51. doi: 10.1038/nature13989 PMC5997177 25470051

[82] Hashimoto D Chow A Noizat C Teo P Beasley MB Leboeuf M. Tissue-resident macrophages self-maintain locally throughout adult life with minimal contribution from circulating monocytes. Immunity (2013) 38(4):792–804. doi: 10.1016/j.immuni.2013.04.004 23601688 PMC3853406

[83] Hoeffel G Chen J Lavin Y Low D Almeida FF See P. C-Myb(+) erythro-myeloid progenitor-derived fetal monocytes give rise to adult tissue-resident macrophages. Immunity (2015) 42(4):665–78. doi: 10.1016/j.immuni.2015.03.011 PMC4545768 25902481

[84] Yona S Kim KW Wolf Y Mildner A Varol D Breker M. Fate mapping reveals origins and dynamics of monocytes and tissue macrophages under homeostasis. Immunity (2013) 38(1):79–91. doi: 10.1016/j.immuni.2012.12.001 23273845 PMC3908543

[85] Wake K Decker K Kirn A Knook DL McCuskey RS Bouwens L. Cell biology and kinetics of Kupffer cells in the liver. Int Rev Cytol (1989) 118:173–229. doi: 10.1016/S0074-7696(08)60875-X 2691426

[86] Bertolino P McCaughan GW Bowen DG. Role of primary intrahepatic T-cell activation in the 'liver tolerance effect'. Immunol Cell Biol (2002) 80(1):84–92. doi: 10.1046/j.0818-9641.2001.01048.x 11869365

[87] Frevert U Usynin I Baer K Klotz C. Nomadic or sessile: can Kupffer cells function as portals for malaria sporozoites to the liver? Cell Microbiol (2006) 8(10):1537–46. doi: 10.1111/j.1462-5822.2006.00777.x 16911567

[88] Mackay IR. Hepatoimmunology: a perspective. Immunol Cell Biol (2002) 80(1):36–44. doi: 10.1046/j.1440-1711.2002.01063.x 11869361

[89] MacPhee PJ Schmidt EE Groom AC. Intermittence of blood flow in liver sinusoids, studied by high-resolution *in vivo* microscopy. Am J Physiol (1995) 269(5 Pt 1):G692–8. doi: 10.1152/ajpgi.1995.269.5.G692 7491960

[90] Markiewski MM DeAngelis RA Lambris JD. Liver inflammation and regeneration: two distinct biological phenomena or parallel pathophysiologic processes? Mol Immunol (2006) 43(1-2):45–56. doi: 10.1016/j.molimm.2005.06.019 16002143

[91] Yang CY Chen JB Tsai TF Tsai YC Tsai CY Liang PH. CLEC4F is an inducible C-type lectin in F4/80-positive cells and is involved in alpha-galactosylceramide presentation in liver. PLoS One (2013) 8(6):e65070. doi: 10.1371/journal.pone.0065070 23762286 PMC3675125

[92] David BA Rezende RM Antunes MM Santos MM Freitas Lopes MA Diniz AB. Combination of mass cytometry and imaging analysis reveals origin, location, and functional repopulation of liver myeloid cells in mice. Gastroenterology (2016) 151(6):1176–91. doi: 10.1053/j.gastro.2016.08.024 27569723

[93] Shan Z Ju C. Hepatic macrophages in liver injury. Front Immunol (2020) 11:322. doi: 10.3389/fimmu.2020.00322 32362892 PMC7180226

[94] Willekens FL Werre JM Kruijt JK Roerdinkholder-Stoelwinder B Groenen-Döpp YA van den Bos AG. Liver Kupffer cells rapidly remove red blood cell-derived vesicles from the circulation by scavenger receptors. Blood (2005) 105(5):2141–5. doi: 10.1182/blood-2004-04-1578 15550489

[95] Shi J Gilbert GE Kokubo Y Ohashi T. Role of the liver in regulating numbers of circulating neutrophils. Blood (2001) 98(4):1226–30. doi: 10.1182/blood.V98.4.1226 11493474

[96] Stark MA Huo Y Burcin TL Morris MA Olson TS Ley K. Phagocytosis of apoptotic neutrophils regulates granulopoiesis via IL-23 and IL-17. Immunity (2005) 22(3):285–94. doi: 10.1016/j.immuni.2005.01.011 15780986

[97] Forlow SB Schurr JR Kolls JK Bagby GJ Schwarzenberger PO Ley K. Increased granulopoiesis through interleukin-17 and granulocyte colony-stimulating factor in leukocyte adhesion molecule-deficient mice. Blood (2001) 98(12):3309–14. doi: 10.1182/blood.V98.12.3309 11719368

[98] Semerad CL Liu F Gregory AD Stumpf K Link DC. G-CSF is an essential regulator of neutrophil trafficking from the bone marrow to the blood. Immunity (2002) 17(4):413–23. doi: 10.1016/S1074-7613(02)00424-7 12387736

[99] Knolle P Schlaak J Uhrig A Kempf P Meyer zum Büschenfelde KH Gerken G. Human Kupffer cells secrete IL-10 in response to lipopolysaccharide (LPS) challenge. J Hepatol (1995) 22(2):226–9. doi: 10.1016/0168-8278(95)80433-1 7790711

[100] Everett ML Collins BH Parker W. Kupffer cells: another player in liver tolerance induction. Liver Transpl (2003) 9(5):498–9. doi: 10.1053/jlts.2003.50092 12740793

[101] Sun Z Wada T Maemura K Uchikura K Hoshino S Diehl AM. Hepatic allograft-derived Kupffer cells regulate T cell response in rats. Liver Transpl (2003) 9(5):489–97. doi: 10.1053/jlts.2003.50091 12740792

[102] Chen Y Liu Z Liang S Luan X Long F Chen J. Role of Kupffer cells in the induction of tolerance of orthotopic liver transplantation in rats. Liver Transpl (2008) 14(6):823–36. doi: 10.1002/lt.21450 18508376

[103] Yan ML Wang YD Tian YF Lai ZD Yan LN. Inhibition of allogeneic T-cell response by Kupffer cells expressing indoleamine 2,3-dioxygenase. World J Gastroenterol (2010) 16(5):636–40. doi: 10.3748/wjg.v16.i5.636 PMC2816279 20128035

[104] Lee J Tam H Adler L Ilstad-Minnihan A Macaubas C Mellins ED. The MHC class II antigen presentation pathway in human monocytes differs by subset and is regulated by cytokines. PLoS One (2017) 12(8):e0183594. doi: 10.1371/journal.pone.0183594 28832681 PMC5568224

[105] Heymann F Hammerich L Storch D Bartneck M Huss S Rüsseler V. Hepatic macrophage migration and differentiation critical for liver fibrosis is mediated by the chemokine receptor C-C motif chemokine receptor 8 in mice. Hepatology (2012) 55(3):898–909. doi: 10.1002/hep.24764 22031018 PMC4533854

[106] Karlmark KR Weiskirchen R Zimmermann HW Gassler N Ginhoux F Weber C. Hepatic recruitment of the inflammatory Gr1+ monocyte subset upon liver injury promotes hepatic fibrosis. Hepatology (2009) 50(1):261–74. doi: 10.1002/hep.22950 19554540

[107] Poulsen KL Cajigas-Du Ross CK Chaney JK Nagy LE. Role of the chemokine system in liver fibrosis: A narrative review. Dig Med Res (2022) 5:30. doi: 10.21037/dmr-21-87 36339901 PMC9632683

[108] Stravitz RT Lee WM. Acute liver failure. Lancet (2019) 394(10201):869–81. doi: 10.1016/S0140-6736(19)31894-X PMC10836844 31498101

[109] Rej R. Aminotransferases in disease. Clin Lab Med (1989) 9(4):667–87. doi: 10.1016/S0272-2712(18)30598-5 2686908

[110] Albano E Rundgren M Harvison PJ Nelson SD Moldeus P. Mechanisms of N-acetyl-p-benzoquinone imine cytotoxicity. Mol Pharmacol (1985) 28(3):306–11. 4033631

[111] Brok J Buckley N Gluud C. Interventions for paracetamol (acetaminophen) overdose. Cochrane Database Syst Rev (2006) 2):CD003328. doi: 10.1002/14651858.CD003328.pub2 16625578

[112] Cirilli I Orlando P Marcheggiani F Dludla PV Silvestri S Damiani E. The protective role of bioactive quinones in stress-induced senescence phenotype of endothelial cells exposed to cigarette smoke extract. Antioxidants (Basel) (2020) 9(10):1008. doi: 10.3390/antiox9101008 33081423 PMC7602940

[113] Dludla PV Mazibuko-Mbeje SE Nyambuya TM Mxinwa V Tiano L Marcheggiani F. The beneficial effects of N-acetyl cysteine (NAC) against obesity associated complications: A systematic review of pre-clinical studies. Pharmacol Res (2019) 146:104332. doi: 10.1016/j.phrs.2019.104332 31254666

[114] Chughlay MF Kramer N Spearman CW Werfalli M Cohen K. N-acetylcysteine for non-paracetamol drug-induced liver injury: a systematic review. Br J Clin Pharmacol (2016) 81(6):1021–9. doi: 10.1111/bcp.12880 PMC4876182 26757427

[115] Ramachandran A Jaeschke H. Acetaminophen hepatotoxicity. Semin Liver Dis (2019) 39(2):221–34. doi: 10.1055/s-0039-1679919 PMC6800176 30849782

[116] Kelly JH Koussayer T He DE Chong MG Shang TA Whisennand HH. An improved model of acetaminophen-induced fulminant hepatic failure in dogs. Hepatology (1992) 15(2):329–35. doi: 10.1002/hep.1840150225 1735538

[117] McGill MR Williams CD Xie Y Ramachandran A Jaeschke H. Acetaminophen-induced liver injury in rats and mice: comparison of protein adducts, mitochondrial dysfunction, and oxidative stress in the mechanism of toxicity. Toxicol Appl Pharmacol (2012) 264(3):387–94. doi: 10.1016/j.taap.2012.08.015 PMC3478469 22980195

[118] Henne-Bruns D Artwohl J Broelsch C Kremer B. Acetaminophen-induced acute hepatic failure in pigs: controversical results to other animal models. Res Exp Med (Berl) (1988) 188(6):463–72. doi: 10.1007/BF01852004 3238177

[119] Barman PK Mukherjee R Prusty BK Suklabaidya S Senapati S Ravindran B. Chitohexaose protects against acetaminophen-induced hepatotoxicity in mice. Cell Death Dis (2016) 7(5):e2224. doi: 10.1038/cddis.2016.131 27171266 PMC4917664

[120] Zhang S Yang N Ni S Li W Xu L Dong P. Pretreatment of lipopolysaccharide (LPS) ameliorates D-GalN/LPS induced acute liver failure through TLR4 signaling pathway. Int J Clin Exp Pathol (2014) 7(10):6626–34. PMC4230075 25400741

[121] Decker K Keppler D. Galactosamine hepatitis: key role of the nucleotide deficiency period in the pathogenesis of cell injury and cell death. Rev Physiol Biochem Pharmacol (1974) 71:77–106. doi: 10.1007/BFb0027661 4375846

[122] Anand R Harry D Holt S Milner P Dashwood M Goodier D. Endothelin is an important determinant of renal function in a rat model of acute liver and renal failure. Gut (2002) 50(1):111–7. doi: 10.1136/gut.50.1.111 PMC1773076 11772977

[123] Arai K Lee K Berthiaume F Tompkins RG Yarmush ML. Intrahepatic amino acid and glucose metabolism in a D-galactosamine-induced rat liver failure model. Hepatology (2001) 34(2):360–71. doi: 10.1053/jhep.2001.26515 11481621

[124] Hefler J Marfil-Garza BA Pawlick RL Freed DH Karvellas CJ Bigam DL. Preclinical models of acute liver failure: a comprehensive review. PeerJ (2021) 9:e12579. doi: 10.7717/peerj.12579 34966588 PMC8667744

[125] Weber LW Boll M Stampfl A. Hepatotoxicity and mechanism of action of haloalkanes: carbon tetrachloride as a toxicological model. Crit Rev Toxicol (2003) 33(2):105–36. doi: 10.1080/713611034 12708612

[126] Shi Z Wakil AE Rockey DC. Strain-specific differences in mouse hepatic wound healing are mediated by divergent T helper cytokine responses. Proc Natl Acad Sci USA (1997) 94(20):10663–8. doi: 10.1073/pnas.94.20.10663 PMC23440 9380692

[127] Rahman TM Hodgson HJ. Animal models of acute hepatic failure. Int J Exp Pathol (2000) 81(2):145–57. doi: 10.1046/j.1365-2613.2000.00144.x PMC2517718 10762442

[128] Du Y Zhang W Qiu H Xiao C Shi J Reid LM. Mouse models of liver parenchyma injuries and regeneration. Front Cell Dev Biol (2022) 10:903740. doi: 10.3389/fcell.2022.903740 35721478 PMC9198899

[129] Heymann F Hamesch K Weiskirchen R Tacke F. The concanavalin A model of acute hepatitis in mice. Lab Anim (2015) 49(1 Suppl):12–20. doi: 10.1177/0023677215572841 25835734

[130] Gantner F Leist M Lohse AW Germann PG Tiegs G. Concanavalin A-induced T-cell-mediated hepatic injury in mice: the role of tumor necrosis factor. Hepatology (1995) 21(1):190–8. doi: 10.1016/0270-9139(95)90428-x 7806154

[131] Mizuhara H Kuno M Seki N Yu WG Yamaoka M Yamashita M. Strain difference in the induction of T-cell activation-associated, interferon gamma-dependent hepatic injury in mice. Hepatology (1998) 27(2):513–9. doi: 10.1002/hep.510270227 9462651

[132] Kakinuma C Takagaki K Yatomi T Nakamura N Nagata S Uemura A. Acute toxicity of an anti-Fas antibody in mice. Toxicol Pathol (1999) 27(4):412–20. doi: 10.1177/019262339902700404 10485821

[133] Tunon MJ Alvarez M Culebras JM Gonzalez-Gallego J. An overview of animal models for investigating the pathogenesis and therapeutic strategies in acute hepatic failure. World J Gastroenterol (2009) 15(25):3086–98. doi: 10.3748/wjg.15.3086 PMC2705730 19575487

[134] Gould AR Kattenbelt JA Lenghaus C Morrissy C Chamberlain T Collins BJ. The complete nucleotide sequence of rabbit haemorrhagic disease virus (Czech strain V351): Use of the polymerase chain reaction to detect replication in Australian vertebrates and analysis of viral population sequence variation. Virus Res (1997) 47(1):7–17. doi: 10.1016/S0168-1702(96)01399-8 9037732

[135] Makino H Togo S Kubota T Morioka D Morita T Kobayashi T. A good model of hepatic failure after excessive hepatectomy in mice. J Surg Res (2005) 127(2):171–6. doi: 10.1016/j.jss.2005.04.029 15916769

[136] Morita T Togo S Kubota T Kamimukai N Nishizuka I Kobayashi T. Mechanism of postoperative liver failure after excessive hepatectomy investigated using a cDNA microarray. J Hepatobiliary Pancreat Surg (2002) 9(3):352–9. doi: 10.1007/s005340200039 12353146

[137] Liu ZC Chang TM. Transdifferentiation of bioencapsulated bone marrow cells into hepatocyte-like cells in the 90% hepatectomized rat model. Liver Transpl (2006) 12(4):566–72. doi: 10.1002/lt.20635 16496278

[138] Gao Y Mu N Xu XP Wang Y. Porcine acute liver failure model established by two-phase surgery and treated with hollow fiber bioartificial liver support system. World J Gastroenterol (2005) 11(35):5468–74. doi: 10.3748/wjg.v11.i35.5468 PMC4320355 16222738

[139] de Groot GH Reuvers CB Schalm SW Boks AL Terpstra OT Jeekel H. A reproducible model of acute hepatic failure by transient ischemia in the pig. J Surg Res (1987) 42(1):92–100. doi: 10.1016/0022-4804(87)90070-9 3807358

[140] Fourneau I Pirenne J Roskams T Yap SH. An improved model of acute liver failure based on transient ischemia of the liver. Arch Surg (2000) 135(10):1183–9. doi: 10.1001/archsurg.135.10.1183 11030876

[141] Andrews TS Atif J Liu JC Perciani CT Ma XZ Thoeni C. Single-cell, single-nucleus, and spatial RNA sequencing of the human liver identifies cholangiocyte and mesenchymal heterogeneity. Hepatol Commun (2022) 6(4):821–40. doi: 10.1002/hep4.1854 PMC8948611 34792289

[142] Saviano A Henderson NC Baumert TF. Single-cell genomics and spatial transcriptomics: Discovery of novel cell states and cellular interactions in liver physiology and disease biology. J Hepatol (2020) 73(5):1219–30. doi: 10.1016/j.jhep.2020.06.004 PMC7116221 32534107

[143] Gerner MY Kastenmuller W Ifrim I Kabat J Germain RN. Histo-cytometry: a method for highly multiplex quantitative tissue imaging analysis applied to dendritic cell subset microanatomy in lymph nodes. Immunity (2012) 37(2):364–76. doi: 10.1016/j.immuni.2012.07.011 PMC3514885 22863836

[144] Giesen C Wang HA Schapiro D Zivanovic N Jacobs A Hattendorf B. Highly multiplexed imaging of tumor tissues with subcellular resolution by mass cytometry. Nat Methods (2014) 11(4):417–22. doi: 10.1038/nmeth.2869 24584193

[145] Goltsev Y Samusik N Kennedy-Darling J Bhate S Hale M Vazquez G. Deep profiling of mouse splenic architecture with CODEX multiplexed imaging. Cell (2018) 174(4):968–81.e15. doi: 10.1016/j.cell.2018.07.010 30078711 PMC6086938

[146] Pirici D Mogoanta L Kumar-Singh S Pirici I Margaritescu C Simionescu C. Antibody elution method for multiple immunohistochemistry on primary antibodies raised in the same species and of the same subtype. J Histochem Cytochem (2009) 57(6):567–75. doi: 10.1369/jhc.2009.953240 PMC2690408 19223296

[147] Porta Siegel T Hamm G Bunch J Cappell J Fletcher JS Schwamborn K. Mass spectrometry imaging and integration with other imaging modalities for greater molecular understanding of biological tissues. Mol Imaging Biol (2018) 20(6):888–901. doi: 10.1007/s11307-018-1267-y 30167993 PMC6244545

[148] Radtke AJ Kandov E Lowekamp B Speranza E Chu CJ Gola A. IBEX: A versatile multiplex optical imaging approach for deep phenotyping and spatial analysis of cells in complex tissues. Proc Natl Acad Sci U S A (2020) 117(52):33455–65. doi: 10.1073/pnas.2018488117 PMC7776876 33376221

[149] Wang J Hossain M Thanabalasuriar A Gunzer M Meininger C Kubes P. Visualizing the function and fate of neutrophils in sterile injury and repair. Science (2017) 358(6359):111–6. doi: 10.1126/science.aam9690 28983053

[150] Wang F Flanagan J Su N Wang LC Bui S Nielson A. RNAscope: a novel in *situ* RNA analysis platform for forMalin-fixed, paraffin-embedded tissues. J Mol Diagn (2012) 14(1):22–9. doi: 10.1016/j.jmoldx.2011.08.002 PMC3338343 22166544

[151] Flores Molina M Fabre T Cleret-Buhot A Soucy G Meunier L Abdelnabi MN. Visualization, quantification, and mapping of immune cell populations in the tumor microenvironment. J Vis Exp (2020) 157:e60740. doi: 10.3791/60740 32281982

[152] Clemens MM McGill MR Apte U. Mechanisms and biomarkers of liver regeneration after drug-induced liver injury. Adv Pharmacol (2019) 85:241–62. doi: 10.1016/bs.apha.2019.03.001 PMC7641498 31307589

[153] Sarhan M Land WG Tonnus W Hugo CP Linkermann A. Origin and consequences of necroinflammation. Physiol Rev (2018) 98(2):727–80. doi: 10.1152/physrev.00041.2016 29465288

[154] Marques PE Vandendriessche S de Oliveira THC Crijns H Lopes ME Blanter M. Inhibition of drug-induced liver injury in mice using a positively charged peptide that binds DNA. Hepatol Commun (2021) 5(10):1737–54. doi: 10.1002/hep4.1759 PMC8485890 34532999

[155] Liu ZX Han D Gunawan B Kaplowitz N. Neutrophil depletion protects against murine acetaminophen hepatotoxicity. Hepatology (2006) 43(6):1220–30. doi: 10.1002/hep.21175 16729305

[156] Wang H Zhang H Wang Y Yang L Wang D. Embelin can protect mice from thioacetamide-induced acute liver injury. BioMed Pharmacother (2019) 118:109360. doi: 10.1016/j.biopha.2019.109360 31545222

[157] Kim EH Wong SW Martinez J. Programmed Necrosis and Disease:We interrupt your regular programming to bring you necroinflammation. Cell Death Differ (2019) 26(1):25–40. doi: 10.1038/s41418-018-0179-3 30349078 PMC6294794

[158] Lindros KO. Zonation of cytochrome P450 expression, drug metabolism and toxicity in liver. Gen Pharmacol (1997) 28(2):191–6. doi: 10.1016/S0306-3623(96)00183-8 9013193

[159] Kennedy RC Smith AK Ropella GEP McGill MR Jaeschke H Hunt CA. Propagation of pericentral necrosis during acetaminophen-induced liver injury: Evidence for early interhepatocyte communication and information exchange. Toxicol Sci (2019) 169(1):151–66. doi: 10.1093/toxsci/kfz029 PMC6484890 30698817

[160] Kang JS Wanibuchi H Morimura K Wongpoomchai R Chusiri Y Gonzalez FJ. Role of CYP2E1 in thioacetamide-induced mouse hepatotoxicity. Toxicol Appl Pharmacol (2008) 228(3):295–300. doi: 10.1016/j.taap.2007.11.010 18374380

[161] Triantafyllou E Pop OT Possamai LA Wilhelm A Liaskou E Singanayagam A. MerTK expressing hepatic macrophages promote the resolution of inflammation in acute liver failure. Gut (2018) 67(2):333–47. doi: 10.1136/gutjnl-2016-313615 PMC5868289 28450389

[162] Richards JA Bucsaiova M Hesketh EE Ventre C Henderson NC Simpson K. Acute liver injury is independent of B cells or immunoglobulin M. PLoS One (2015) 10(9):e0138688. doi: 10.1371/journal.pone.0138688 26406765 PMC4583453

[163] Devisscher L Scott CL Lefere S Raevens S Bogaerts E Paridaens A. Non-alcoholic steatohepatitis induces transient changes within the liver macrophage pool. Cell Immunol (2017) 322:74–83. doi: 10.1016/j.cellimm.2017.10.006 29111158

[164] Dambach DM Watson LM Gray KR Durham SK Laskin DL. Role of CCR2 in macrophage migration into the liver during acetaminophen-induced hepatotoxicity in the mouse. Hepatology (2002) 35(5):1093–103. doi: 10.1053/jhep.2002.33162 11981759

[165] Dou L Shi X He X Gao Y. Macrophage phenotype and function in liver disorder. Front Immunol (2019) 10:3112. doi: 10.3389/fimmu.2019.03112 32047496 PMC6997484

[166] Nakamoto N Kanai T. Role of toll-like receptors in immune activation and tolerance in the liver. Front Immunol (2014) 5:221. doi: 10.3389/fimmu.2014.00221 24904576 PMC4032908

[167] Schwabe RF Brenner DA. Mechanisms of Liver Injury. I. TNF-alpha-induced liver injury: role of IKK, JNK, and ROS pathways. Am J Physiol Gastrointest Liver Physiol (2006) 290(4):G583–9. doi: 10.1152/ajpgi.00422.2005 16537970

[168] Connolly MK Bedrosian AS Mallen-St Clair J Mitchell AP Ibrahim J Stroud A. In liver fibrosis, dendritic cells govern hepatic inflammation in mice via TNF-alpha. J Clin Invest (2009) 119(11):3213–25. doi: 10.1172/jci37581 PMC2769179 19855130

[169] Yang YM Seki E. TNFα in liver fibrosis. Curr Pathobiol Rep (2015) 3(4):253–61. doi: 10.1007/s40139-015-0093-z PMC4693602 26726307

[170] Huang YH Shi MN Zheng WD Zhang LJ Chen ZX Wang XZ. Therapeutic effect of interleukin-10 on CCl4-induced hepatic fibrosis in rats. World J Gastroenterol (2006) 12(9):1386–91. doi: 10.3748/wjg.v12.i9.1386 PMC4124315 16552806

[171] Louis H Van Laethem JL Wu W Quertinmont E Degraef C Van den Berg K. Interleukin-10 controls neutrophilic infiltration, hepatocyte proliferation, and liver fibrosis induced by carbon tetrachloride in mice. Hepatology (1998) 28(6):1607–15. doi: 10.1002/hep.510280621 9828225

[172] Thompson K Maltby J Fallowfield J McAulay M Millward-Sadler H Sheron N. Interleukin-10 expression and function in experimental murine liver inflammation and fibrosis. Hepatology (1998) 28(6):1597–606. doi: 10.1002/hep.510280620 9828224

[173] Alegre F Pelegrin P Feldstein AE. Inflammasomes in liver fibrosis. Semin Liver Dis (2017) 37(2):119–27. doi: 10.1055/s-0037-1601350 28564720

[174] Dinarello CA. Immunological and inflammatory functions of the interleukin-1 family. Annu Rev Immunol (2009) 27:519–50. doi: 10.1146/annurev.immunol.021908.132612 19302047

[175] Gieling RG Wallace K Han YP. Interleukin-1 participates in the progression from liver injury to fibrosis. Am J Physiol Gastrointest Liver Physiol (2009) 296(6):G1324–31. doi: 10.1152/ajpgi.90564.2008 PMC2697947 19342509

[176] Karlmark KR Zimmermann HW Roderburg C Gassler N Wasmuth HE Luedde T. The fractalkine receptor CX₃CR1 protects against liver fibrosis by controlling differentiation and survival of infiltrating hepatic monocytes. Hepatology (2010) 52(5):1769–82. doi: 10.1002/hep.23894 21038415

[177] Graubardt N Vugman M Mouhadeb O Caliari G Pasmanik-Chor M Reuveni D. Ly6C(hi) monocytes and their macrophage descendants regulate neutrophil function and clearance in acetaminophen-induced liver injury. Front Immunol (2017) 8:626. doi: 10.3389/fimmu.2017.00626 28620383 PMC5451509

[178] Nguyen NT Umbaugh DS Smith S Adelusi OB Sanchez-Guerrero G Ramachandran A. Dose-dependent pleiotropic role of neutrophils during acetaminophen-induced liver injury in male and female mice. Arch Toxicol (2023) 97(5):1397–412. doi: 10.1007/s00204-023-03478-4 PMC10680445 36928416

[179] Guilliams M Mildner A Yona S. Developmental and functional heterogeneity of monocytes. Immunity (2018) 49(4):595–613. doi: 10.1016/j.immuni.2018.10.005 30332628

[180] Olingy CE San Emeterio CL Ogle ME Krieger JR Bruce AC Pfau DD. Non-classical monocytes are biased progenitors of wound healing macrophages during soft tissue injury. Sci Rep (2017) 7(1):447. doi: 10.1038/s41598-017-00477-1 28348370 PMC5428475

[181] Alkhani A Levy CS Tsui M Rosenberg KA Polovina K Mattis AN. Ly6c(Lo) non-classical monocytes promote resolution of rhesus rotavirus-mediated perinatal hepatic inflammation. Sci Rep (2020) 10(1):7165. doi: 10.1038/s41598-020-64158-2 32346042 PMC7188847

[182] Font-Burgada J Shalapour S Ramaswamy S Hsueh B Rossell D Umemura A. Hybrid periportal hepatocytes regenerate the injured liver without giving rise to cancer. Cell (2015) 162(4):766–79. doi: 10.1016/j.cell.2015.07.026 PMC4545590 26276631

[183] Ge Y Huang M Yao YM. Efferocytosis and its role in inflammatory disorders. Front Cell Dev Biol (2022) 10:839248. doi: 10.3389/fcell.2022.839248 35281078 PMC8913510

[184] Horst AK Tiegs G Diehl L. Contribution of macrophage efferocytosis to liver homeostasis and disease. Front Immunol (2019) 10:2670. doi: 10.3389/fimmu.2019.02670 31798592 PMC6868070

[185] Triantafyllou E Woollard KJ McPhail MJW Antoniades CG Possamai LA. The role of monocytes and macrophages in acute and acute-on-chronic liver failure. Front Immunol (2018) 9:2948. doi: 10.3389/fimmu.2018.02948 30619308 PMC6302023

[186] Trahtemberg U Mevorach D. Apoptotic cells induced signaling for immune homeostasis in macrophages and dendritic cells. Front Immunol (2017) 8:1356. doi: 10.3389/fimmu.2017.01356 29118755 PMC5661053

[187] Jiang Y Tang Y Hoover C Kondo Y Huang D Restagno D. Kupffer cell receptor CLEC4F is important for the destruction of desialylated platelets in mice. Cell Death Differ (2021) 28(11):3009–21. doi: 10.1038/s41418-021-00797-w PMC8564511 33993195

[188] Zeng Z Surewaard BG Wong CH Geoghegan JA Jenne CN Kubes P. CRIg functions as a macrophage pattern recognition receptor to directly bind and capture blood-borne gram-positive bacteria. Cell Host Microbe (2016) 20(1):99–106. doi: 10.1016/j.chom.2016.06.002 27345697

[189] Zhang M Xu S Han Y Cao X. Apoptotic cells attenuate fulminant hepatitis by priming Kupffer cells to produce interleukin-10 through membrane-bound TGF-beta. Hepatology (2011) 53(1):306–16. doi: 10.1002/hep.24029 21140375

[190] Stutchfield BM Antoine DJ Mackinnon AC Gow DJ Bain CC Hawley CA. CSF1 restores innate immunity after liver injury in mice and serum levels indicate outcomes of patients with acute liver failure. Gastroenterology (2015) 149(7):1896–909.e14. doi: 10.1053/j.gastro.2015.08.053 26344055 PMC4672154

[191] Rolando N Wade J Davalos M Wendon J Philpott-Howard J Williams R. The systemic inflammatory response syndrome in acute liver failure. Hepatology (2000) 32(4 Pt 1):734–9. doi: 10.1053/jhep.2000.17687 11003617

[192] Rantakari P Patten DA Valtonen J Karikoski M Gerke H Dawes H. Stabilin-1 expression defines a subset of macrophages that mediate tissue homeostasis and prevent fibrosis in chronic liver injury. Proc Natl Acad Sci USA (2016) 113(33):9298–303. doi: 10.1073/pnas.1604780113 PMC4995933 27474165

[193] Van Rooijen N Sanders A. Kupffer cell depletion by liposome-delivered drugs: comparative activity of intracellular clodronate, propamidine, and ethylenediaminetetraacetic acid. Hepatology (1996) 23(5):1239–43. doi: 10.1002/hep.510230544 8621159

[194] Zhao S Jiang J Jing Y Liu W Yang X Hou X. The concentration of tumor necrosis factor-α determines its protective or damaging effect on liver injury by regulating Yap activity. Cell Death Dis (2020) 11(1):70. doi: 10.1038/s41419-020-2264-z 31988281 PMC6985193

[195] Yamada Y Fausto N. Deficient liver regeneration after carbon tetrachloride injury in mice lacking type 1 but not type 2 tumor necrosis factor receptor. Am J Pathol (1998) 152(6):1577–89. PMC1858451 9626061

[196] You Q Holt M Yin H Li G Hu CJ Ju C. Role of hepatic resident and infiltrating macrophages in liver repair after acute injury. Biochem Pharmacol (2013) 86(6):836–43. doi: 10.1016/j.bcp.2013.07.006 PMC3863645 23876342

[197] Miura A Hosono T Seki T. Macrophage potentiates the recovery of liver zonation and metabolic function after acute liver injury. Sci Rep (2021) 11(1):9730. doi: 10.1038/s41598-021-88989-9 33958644 PMC8102573

[198] Pinzani M Macias-Barragan J. Update on the pathophysiology of liver fibrosis. Expert Rev Gastroenterol Hepatol (2010) 4(4):459–72. doi: 10.1586/egh.10.47 20678019

[199] Dooley S ten Dijke P. TGF-beta in progression of liver disease. Cell Tissue Res (2012) 347(1):245–56. doi: 10.1007/s00441-011-1246-y PMC3250614 22006249

[200] Giannelli G Mikulits W Dooley S Fabregat I Moustakas A ten Dijke P. The rationale for targeting TGF-beta in chronic liver diseases. Eur J Clin Invest (2016) 46(4):349–61. doi: 10.1111/eci.12596 26823073

[201] Date M Matsuzaki K Matsushita M Sakitani K Shibano K Okajima A. Differential expression of transforming growth factor-beta and its receptors in hepatocytes and nonparenchymal cells of rat liver after CCl4 administration. J Hepatol (1998) 28(4):572–81. doi: 10.1016/S0168-8278(98)80280-8 9566825

[202] Date M Matsuzaki K Matsushita M Tahashi Y Furukawa F Inoue K. Modulation of transforming growth factor beta function in hepatocytes and hepatic stellate cells in rat liver injury. Gut (2000) 46(5):719–24. doi: 10.1136/gut.46.5.719 PMC1727924 10764719

[203] McMillin M Galindo C Pae HY Frampton G Di Patre PL Quinn M. Gli1 activation and protection against hepatic encephalopathy is suppressed by circulating transforming growth factor beta1 in mice. J Hepatol (2014) 61(6):1260–6. doi: 10.1016/j.jhep.2014.07.015 PMC4253574 25046848

[204] Coelho I Duarte N Barros A Macedo MP Penha-Goncalves C. Trem-2 promotes emergence of restorative macrophages and endothelial cells during recovery from hepatic tissue damage. Front Immunol (2020) 11:616044. doi: 10.3389/fimmu.2020.616044 33628208 PMC7897679

[205] Perugorria MJ Esparza-Baquer A Oakley F Labiano I Korosec A Jais A. Non-parenchymal TREM-2 protects the liver from immune-mediated hepatocellular damage. Gut (2019) 68(3):533–46. doi: 10.1136/gutjnl-2017-314107 PMC6580759 29374630

[206] Bronte V Zanovello P. Regulation of immune responses by L-arginine metabolism. Nat Rev Immunol (2005) 5(8):641–54. doi: 10.1038/nri1668 16056256

[207] Gordon S. Alternative activation of macrophages. Nat Rev Immunol (2003) 3(1):23–35. doi: 10.1038/nri978 12511873

[208] Arribas-López E Zand N Ojo O Snowden MJ Kochhar T. The effect of amino acids on wound healing: A systematic review and meta-analysis on arginine and glutamine. Nutrients (2021) 13(8):2498. doi: 10.3390/nu13082498 34444657 PMC8399682

[209] Breen FN Hume DA Weidemann MJ. Interactions among granulocyte-macrophage colony-stimulating factor, macrophage colony-stimulating factor, and IFN-gamma lead to enhanced proliferation of murine macrophage progenitor cells. J Immunol (1991) 147(5):1542–7. doi: 10.4049/jimmunol.147.5.1542 1908878

[210] Hume DA MacDonald KP. Therapeutic applications of macrophage colony-stimulating factor-1 (CSF-1) and antagonists of CSF-1 receptor (CSF-1R) signaling. Blood (2012) 119(8):1810–20. doi: 10.1182/blood-2011-09-379214 22186992

[211] Hume DA Pavli P Donahue RE Fidler IJ. The effect of human recombinant macrophage colony-stimulating factor (CSF-1) on the murine mononuclear phagocyte system *in vivo* . J Immunol (1988) 141(10):3405–9. doi: 10.4049/jimmunol.141.10.3405 3053899

[212] Keshvari S Genz B Teakle N Caruso M Cestari MF Patkar OL. Therapeutic potential of macrophage colony-stimulating factor in chronic liver disease. Dis Model Mech (2022) 15(4):dmm049387. doi: 10.1242/dmm.049387 35169835 PMC9044210

[213] White GE Greaves DR. Fractalkine: a survivor's guide: chemokines as antiapoptotic mediators. Arterioscler Thromb Vasc Biol (2012) 32(3):589–94. doi: 10.1161/ATVBAHA.111.237412 22247260

[214] Jones BA Beamer M Ahmed S. Fractalkine/CX3CL1: a potential new target for inflammatory diseases. Mol Interv (2010) 10(5):263–70. doi: 10.1124/mi.10.5.3 PMC3002219 21045240

[215] D'Haese JG Demir IE Friess H Ceyhan GO. Fractalkine/CX3CR1: why a single chemokine-receptor duo bears a major and unique therapeutic potential. Expert Opin Ther Targets (2010) 14(2):207–19. doi: 10.1517/14728220903540265 20055718

[216] Devalaraja RM Nanney LB Du J Qian Q Yu Y Devalaraja MN. Delayed wound healing in CXCR2 knockout mice. J Invest Dermatol (2000) 115(2):234–44. doi: 10.1046/j.1523-1747.2000.00034.x PMC2664868 10951241

[217] Hossain M Kubes P. Innate immune cells orchestrate the repair of sterile injury in the liver and beyond. Eur J Immunol (2019) 49(6):831–41. doi: 10.1002/eji.201847485 31001813

[218] Rosales C. Neutrophil: A cell with many roles in inflammation or several cell types? Front Physiol (2018) 9:113. doi: 10.3389/fphys.2018.00113 29515456 PMC5826082

[219] Silvestre-Roig C Hidalgo A Soehnlein O. Neutrophil heterogeneity: implications for homeostasis and pathogenesis. Blood (2016) 127(18):2173–81. doi: 10.1182/blood-2016-01-688887 27002116

[220] Bouwens L Baekeland M De Zanger R Wisse E. Quantitation, tissue distribution and proliferation kinetics of Kupffer cells in normal rat liver. Hepatology (1986) 6(4):718–22. doi: 10.1002/hep.1840060430 3733004

[221] Itoh Y Okanoue T Morimoto M Nagao Y Mori T Hori N. Functional heterogeneity of rat liver macrophages: interleukin-1 secretion and Ia antigen expression in contrast with phagocytic activity. Liver (1992) 12(1):26–33. doi: 10.1111/j.1600-0676.1992.tb00551.x 1564982

[222] Sleyster EC Knook DL. Relation between localization and function of rat liver Kupffer cells. Lab Invest (1982) 47(5):484–90. 6182391

[223] McCuskey RS. Morphological mechanisms for regulating blood flow through hepatic sinusoids. Liver (2000) 20(1):3–7. doi: 10.1034/j.1600-0676.2000.020001003.x 10726955

[224] Tacke F Zimmermann HW. Macrophage heterogeneity in liver injury and fibrosis. J Hepatol (2014) 60(5):1090–6. doi: 10.1016/j.jhep.2013.12.025 24412603

[225] Ramachandran P Iredale JP Fallowfield JA. Resolution of liver fibrosis: basic mechanisms and clinical relevance. Semin Liver Dis (2015) 35(2):119–31. doi: 10.1055/s-0035-1550057 25974898

[226] Antoniades CG Quaglia A Taams LS Mitry RR Hussain M Abeles R. Source and characterization of hepatic macrophages in acetaminophen-induced acute liver failure in humans. Hepatology (2012) 56(2):735–46. doi: 10.1002/hep.25657 22334567

[227] Blériot C Dupuis T Jouvion G Eberl G Disson O Lecuit M. Liver-resident macrophage necroptosis orchestrates type 1 microbicidal inflammation and type-2-mediated tissue repair during bacterial infection. Immunity (2015) 42(1):145–58. doi: 10.1016/j.immuni.2014.12.020 25577440

[228] Guidotti LG Inverso D Sironi L Di Lucia P Fioravanti J Ganzer L. Immunosurveillance of the liver by intravascular effector CD8(+) T cells. Cell (2015) 161(3):486–500. doi: 10.1016/j.cell.2015.03.005 25892224 PMC11630812

[229] Fujita T Narumiya S. Roles of hepatic stellate cells in liver inflammation: a new perspective. Inflammation Regen (2016) 36:1. doi: 10.1186/s41232-016-0005-6 PMC5721720 29259674

[230] Cai X Wang J Wang J Zhou Q Yang B He Q. Intercellular crosstalk of hepatic stellate cells in liver fibrosis: New insights into therapy. Pharmacol Res (2020) 155:104720. doi: 10.1016/j.phrs.2020.104720 32092405

[231] Gandhi CR. Hepatic stellate cell activation and pro-fibrogenic signals. J Hepatol (2017) 67(5):1104–5. doi: 10.1016/j.jhep.2017.06.001 PMC5679016 28939135

[232] Wen Y Lambrecht J Ju C Tacke F. Hepatic macrophages in liver homeostasis and diseases-diversity, plasticity and therapeutic opportunities. Cell Mol Immunol (2021) 18(1):45–56. doi: 10.1038/s41423-020-00558-8 33041338 PMC7852525

[233] Cai B Dongiovanni P Corey KE Wang X Shmarakov IO Zheng Z. Macrophage merTK promotes liver fibrosis in nonalcoholic steatohepatitis. Cell Metab (2020) 31(2):406–21.e7. doi: 10.1016/j.cmet.2019.11.013 31839486 PMC7004886

[234] Odenwald MA Turner JR. Intestinal permeability defects: is it time to treat? Clin Gastroenterol Hepatol (2013) 11(9):1075–83. doi: 10.1016/j.cgh.2013.07.001 PMC3758766 23851019

[235] Friedman SL Arthur MJ. Activation of cultured rat hepatic lipocytes by Kupffer cell conditioned medium. Direct enhancement of matrix synthesis and stimulation of cell proliferation via induction of platelet-derived growth factor receptors. J Clin Invest (1989) 84(6):1780–5. doi: 10.1172/jci114362 PMC304055 2556445

[236] Nieto N. Oxidative-stress and IL-6 mediate the fibrogenic effects of [corrected] Kupffer cells on stellate cells. Hepatology (2006) 44(6):1487–501. doi: 10.1002/hep.21427 17133487

[237] Cheng D Chai J Wang H Fu L Peng S Ni X. Hepatic macrophages: Key players in the development and progression of liver fibrosis. Liver Int (2021) 41(10):2279–94. doi: 10.1111/liv.14940 33966318

